# A Stable Hexaazaoctacene Cruciform *σ*‐Dimer

**DOI:** 10.1002/advs.202202710

**Published:** 2022-07-27

**Authors:** Steffen Maier, Fabian Jester, Marvin T. Hoffmann, Frank Rominger, Jan Freudenberg, Andreas Dreuw, Uwe H. F. Bunz

**Affiliations:** ^1^ Organisch‐Chemisches Institut Ruprecht‐Karls‐Universität Heidelberg Im Neuenheimer Feld 270 69120 Heidelberg Germany; ^2^ Interdisziplinäres Zentrum für Wissenschaftliches Rechnen Universität Heidelberg Im Neuenheimer Feld 205A 69120 Heidelberg Germany; ^3^ Physikalisch‐Chemisches Institut Universität Heidelberg Im Neuenheimer Feld 253 69120 Heidelberg Germany; ^4^ Centre for Advanced Materials (CAM) Ruprecht‐Karls‐Universität Heidelberg Im Neuenheimer Feld 225 69120 Heidelberg Germany

**Keywords:** acenes, azaoctacene, biacenyl, heteroacene, octacene, stabilization

## Abstract

Buchwald‐Hartwig coupling of a triisopropylsilyl (TIPS)‐ethynylated dibromo‐*N,N*'‐dihydrotetraazapentacene with 1,4‐bis(TIPS‐ethynyl)‐2,3‐diaminonaphthalene furnishes a dihydrohexaazaoctacene. Its oxidation with MnO_2_ results in a 7,7'‐bi(hexaazaoctacenyl). In addition to eight TIPS‐ethynyl groups, the bioctacene motif protects the azaoctacene subunits. The biazaoctacenyl displays a *τ*_1/2_ of > 5 d in dilute solution under ambient conditions. In the crystalline state it is persistent for > 10 months.

## Graphical abstract

In contrast to a triisopropylsilyl (TIPS)‐ethynylated hexaazaoctacene, its *σ*‐dimer, a bihexaazaoctacenyl, is persistent in hexane and stable as a crystalline solid.

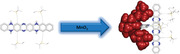

## 1 Introduction

**Figure 1:**
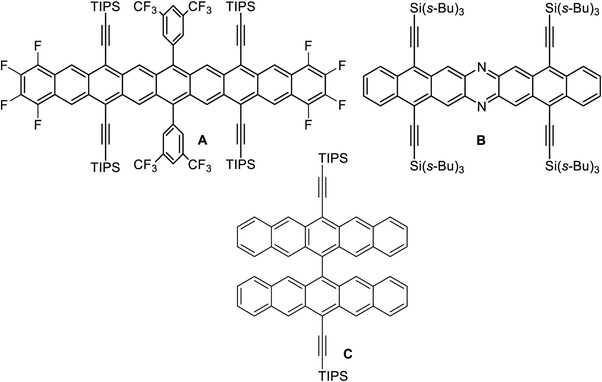
Structures of A) Anthony's nonacene, B) Bunz’ diazaheptacene, and C) Wu's bipentacenyl.^[^ ^10^, ^15^, ^17^ ^]^

Herein, we report a hexaazaoctacene, stabilized by *σ*‐cruciform‐dimerization. Acenes,^[^ ^1^ ^]^ fundamentally important, lose solubility and stability beyond pentacene.^[^ ^2^ ^]^ The larger acenes dimerize^[^ ^3^ ^]^ or form oxygen^[^ ^4^ ^]^ adducts. Confinement on surfaces^[^ ^5^ ^]^ or in matrices^[^ ^6^ ^]^ allows to study the parents; the largest one studied to date is dodecacene, as reported by Peña, Moresco et al.^[^ ^7^ ^]^ Soluble, processable, and persistent derivatives require multiple silylethynyls (R_3_Si—C≡C—) culminating in the preparation of hexacenes,^[^ ^8^ ^]^ heptacenes,^[^ ^9^ ^]^ and a nonacene^[^ ^10^ ^]^ (see **Figure** **1**A) pioneered by Anthony et al.^[^ ^11^ ^]^ In azaacenes^[^ ^12^ ^]^ one or multiple CH— groups of the acenes are replaced by pyridine nitrogen atoms—representatives with one or two embedded pyrazine rings are most easily generated.^[^ ^13^ ^]^ Oxidative stability improves as azaacenes are electron poor and [4+4]‐cycloadditions do not occur at the pyrazine rings, yet azaacenes with more than six nitrogen atoms spontaneously react to their *N,N*'‐dihydro‐derivatives, possibly by oxidation of O_2_.^[^ ^14^ ^]^ **B**, currently the largest azaacene, slowly dimerizes in solution.^[^ ^15^ ^]^ Longer linear azaarenes are known but require additional Clar sextets for stabilization.^[^ ^16^ ^]^

Cruciform dimers of the higher acenes are rare. Most of them are bianthryls,^[^ ^18^ ^]^ and only a handful of bitetracenyls^[^ ^19^ ^]^ or bipentacenyls^[^ ^20^ ^]^ are known, a few of which are triisopropylsilyl (TIPS)‐ethynylated. Pentacene dimer **C**, reported by Wu et al., is more stable than TIPS‐pentacene with respect to photooxidation,^[^ ^17^ ^]^ indicating that acene‐based cruciform scaffold could stabilize the higher (aza)acenes.

## 2 Results and Discussion

**Scheme 1:**
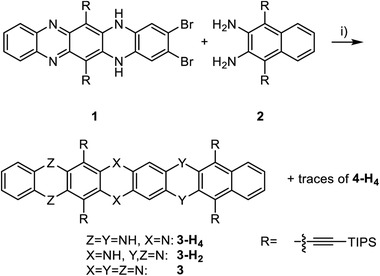
Synthesis of precursor **3**‐H_2_. Conditions: i) Cs_2_CO_3_, RuPhos Pd G1 (10 mol%), toluene, 140 °C, 20 h, 53% (**3**‐H_2_).

**Scheme 2:**
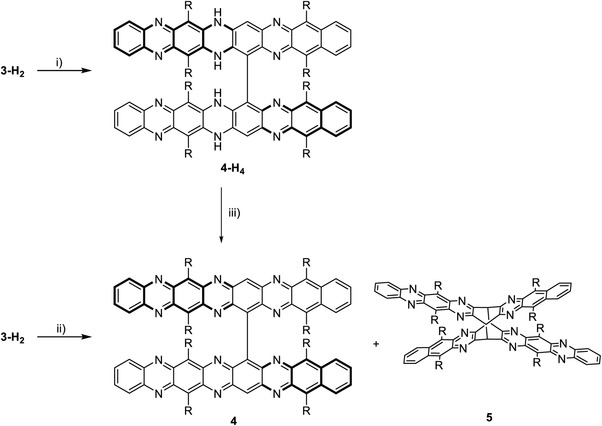
Dimerization of **3**‐H_2_. i) Cs_2_CO_3_, RuPhos Pd G1 (10 mol%), toluene, 140 °C, 20 h, **4**‐H_4_: 40% – **4** and **5** were not observed. ii) MnO_2_, CHCl_3_, 60°C, 1 h. Yields: **4**: 24%, **5**: 53%. iii) MnO_2_, DCM, r.t., 1 h. Yields: **4**: 98%. One enantiomer of **4** depicted for clarity.

Buchwald‐Hartwig^[^ ^21^ ^]^ (BH) coupling of **1** (mixture of tautomers, see Supporting Information for synthesis) with diamine **2** (**Scheme** **1**) yielded **3**‐H_2_, isolated in 53% after column chromatography. Electron‐rich **3**‐H_4_ concomitantly formed in varying yields (see Figure S29, Supporting Information, for crystal structure) and slowly oxidized to **3‐**H_2_ under air. Oxidation exclusively occurred at the eastern phenazine moiety according to NMR. We also observed traces of dimeric **4**‐H_4_ generated in situ under the specific BH conditions employed. Without the diamine, **3**‐H_2_ dimerized to **4**‐H_4_ in the presence of 10 mol% RuPhos Pd G1 in 40% (**Scheme** **2**). Pd(PPh_3_)_4_, Pd_2_(dba)_3_/DavePhos, Brettphos Pd G3 or (PPh_3_)_2_PdCl_2_ did not convert the starting material.

Treatment of **4‐**H_4_ with MnO_2_ at room temperature in DCM results in nearly quantitative formation of **4**. If **3**‐H_2_ was directly oxidized with MnO_2_ at 60 °C, we isolated brown **4** (24%) and the butterfly dimer of **4**, **5** (53%, Figure S28, Supporting Information, crystal structure) after column chromatography. During purification, **4** partially reduces itself on silica gel. We isolated a mixture of **4**‐H_4_ and **4,** which we subsequently oxidized with MnO_2_ to yield the pure compound. Formation of **4** and **5** was accompanied by that of various other products, which we were not able to isolate. At room temperature yields for **4** and **5** (direct oxidation of **3‐**H_2_) changed to 4% and 63%, respectively. **4** and **5** were also observed when **3‐**H_4_ was the starting material or PbO_2_ was employed as oxidant. In contrast to highly reactive hexaazaoctacene **3**, which must have formed in situ as it is the precursor to the formal [4+4] dimer **5**, bioctacenyl **4** is persistent and survives chromatography on silica in air. In the proton NMR, the resonance of the central protons on the octacene backbone is found between 9.5 and 10.0 ppm—it is significantly broadened. This is attributed to the diradical character of **4**, as also a weak signal in the EPR spectrum is observed (Figure S27, Supporting Information).

**Figure 2:**
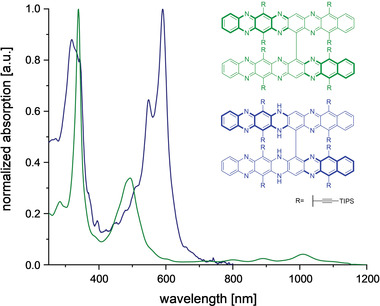
Normalized absorption spectra of oxidized octacene dimer **4** and its corresponding NH‐species **4‐**H_4_ in DCM.

**Figure** **2** compares the absorption spectra (DCM) of **4** and **4**‐H_4_. The oxidation to **4** results in a red shift of 419 nm (7019 cm^−1^) when going from **4**‐H_4_ (*λ*_max,abs_ = 591 nm) to **4** (*λ*_max,abs_ = 1010 nm). The typical vibronic acene finger structure is observed in the near infra‐red—its shoulder at long wavelengths is indicative of its diradical character.^[^ ^22^ ^]^

As expected, the p‐band of **4** is red‐shifted by 151 nm (1740 cm^−1^) compared to that of heptacene B (*λ*_max,abs_  =  859 nm, n‐hexane).^[^ ^15^ ^]^ In contrast to emissive **4**‐H_4_ (*λ*_max,em_  =  635 nm, DCM) and as expected for an octacene and systems larger than heptacenes, excitation at 492 nm (see Figures S12 and S13, Supporting Information) does not yield any appreciable fluorescence of **4** in the visible/NIR range. The *π*‐system is thus fully oxidized and does not contain smaller acene fragments.

Comparing the optical spectra of **3**‐H_2_ to that of **4**‐H_4_ (Figure S2, Supporting Information), we observe only a minute red shift indicating deconjugation of the perpendicular chromophores. Therefore, we expect similar absorption profiles of the reactive and elusive hexaazaoctacene **3** and of **4**.

Quantum chemical calculations (TDDFT, CAM‐B3LYP/pcseg‐1/PCM(DCM)) predict absorption bands at 1044 nm (see Figure S9, Supporting Information) for **4** with an oscillator strength of *f* = 0.07. As a consequence, the observed optical gap (1.23 eV) lies within the range of error for the calculated HOMO LUMO gap (0.96 eV, DFT, Gaussian16^[^ ^23^ ^]^ B3LYP/def2‐TZVP). The latter is similar to that of its hypothetical monomer **3** (1.07 eV) although *σ*‐dimerization destabilizes FMO energy levels.

NICS(1)^[^ ^24^ ^]^ values (see Figure S3, Supporting Information) of **4** range between ‐7.8 to ‐13.4 ppm with the lowest values at the ends of the aromatic backbone, typical for acenes and azaacenes. NICS and AICD^[^ ^25^ ^]^ (Figures S3 and S4, Supporting Information) calculations support complete delocalization of the respective *π*‐systems.

In cyclic voltammograms, **4** displays five redox events, corresponding to consecutive reductions in DCM. The first reduction event was observed at *E* ^0/−^ = ‐0.48 V (vs ferrocene). **4** is more easily reduced than electron‐deficient tetrabromotetraazapentacene (*E* ^0/−^ = ‐0.7 V)^[^ ^26^ ^]^ and thus its radical ion should be air‐stable. During the measurement, we observed fouling on the electrodes, which results in a low‐quality spectrum. As expected, the more electron‐rich **4**‐H_4_ is less easily reduced (*E* ^0/−^ = ‐1.48 V) (**Table** **1**).

**Table 1:** Experimental and calculated (gas‐phase) properties of monomers **3** and **3**‐H_2_ and dimers **4** and **4**‐H_4_ in DCM

Compound	*_*λ*_*_max, abs_ [nm]	*E* ^(0/‐)^ [V]^a)^	Ionization potential/ HOMO [eV] ^b)^meas._CV_/^c)^meas._UV_ /^d)^ calcd	Electron affinity/ LUMO [eV] ^e)^meas./^d)^calcd.
**4**	1010	−0.48	−4.96/−5.85/−5.18	−4.62/−4.22
**4**‐H_4_	591	−1.48	−4.73/−5.72/−5.28	−3.62/−3.19
**3**	–	–	–/–/−5.34	–/−4.27
**3**‐H_2_	593	−1.53	−4.73/−5.66/−5.28	−3.57/−3.12

^a)^ First reduction potentials from cyclic voltammetry (CV) in DCM at room temperature with Bu_4_NPF_6_ as the electrolyte against Fc/Fc^+^ as an internal standard (−5.10 eV) at 0.2 V/s;^[^ ^27^ ^]^

^b)^ Ionization potential_meas,CV_ =  −e×(5.1 V+*E* ^(0/+)^)

^c)^ Ionization potential_meas__._ =  electron affinity_meas._ – gap_meas._;

^d)^ Obtained from DFT calculations (Gaussian 16^[^ ^23^ ^]^ B3LYP/ def2‐TZVP, using the geometry of the crystal structure);

^e)^ Electron affinity_meas__._ =  −e×(5.1 V+*E* ^(0/−)^).

**Figure 3:**
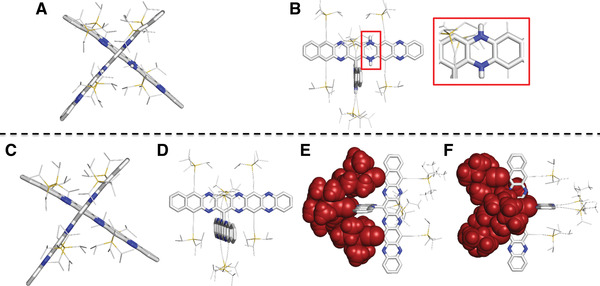
X‐ray single‐crystal structures of A) **4‐**H_4_ front view; B) **4‐**H_4_ top view; C) **4** front view; D) **4** top view; E) **4** side view, shielding TIPS‐ethynyl groups were shown as van‐der‐Waals radii; F) **4** side view, shielding TIPS‐ethynyl groups are shown as van‐der‐Waals radii.

Single crystals of **4**‐H_4_, **4** and **5** were grown by diffusion of MeOH into chloroform solutions (**Figure** **3** and Figures S27 and S28, Supporting Information). **4‐**H_4_ and **4** crystallize as a mixture of atropisomers (enantiomers) in the space group C2/c. Bond lengths and bond angles are in good agreement with the calculated values. The twist angles obtained by X‐ray diffraction (84.5° and 85.3°) differ from the calculated values (65.8°, 68.5°) due to less steric hindrance when using TMS (trimethylsilyl) groups instead of TIPS groups for the calculated geometry.

The dihydropyrazine character is evident for **4**‐H_4_ (Figure 3B) and absent in **4**. *σ*‐dimerization results in a cruciform structure in which four of the R_3_Si‐CC‐substituents protect the connected acene units from [4+4] dimerization by sheer steric bulk. Oxidation of **4** should be of no concern, it is electron poor. Single crystals of **4** are stable for more than two weeks under ambient conditions according to their unaltered diffraction images.

**Figure 4:**
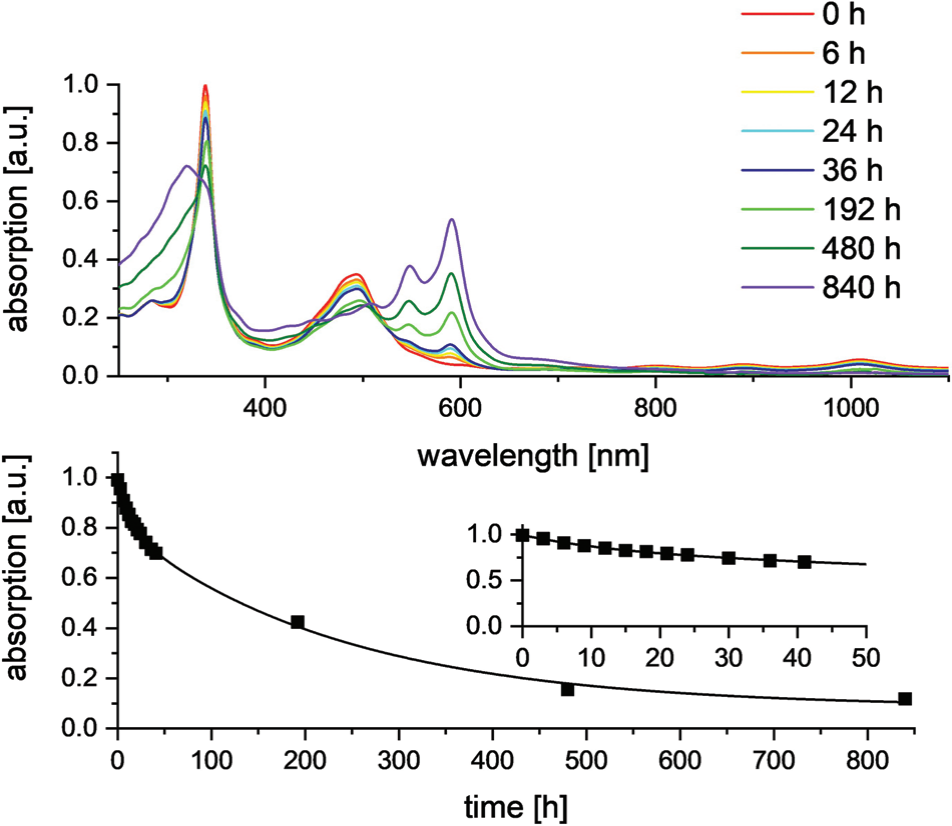
Change in UV/vis absorption intensity of **4** under ambient conditions (top). Time dependent decrease of the absorption band at 1010 nm of **4** under ambient conditions in DCM solution. For clarity reasons, intensity values were fitted with an exponential fit.

**Figure** **4**, bottom, compares the stabilities of dilute solutions of **4** in DCM monitored at 1010 nm. The bioctacenyl displays a half‐life of more than 5 (!) d under ambient (air, exposure to daylight) conditions. Even after 35 d, weak absorption of the absorption band at 1010 nm is visible. The octacene dimer **4** reduces itself slowly to the corresponding NH‐species **4**‐H_4_ supported by the comparison of their absorption spectra (see Figure S23, Supporting Information). A mechanism of the reduction was proposed by Zhang et al. relying on the diradical character of the larger heteroacenes with delocalization of the unpaired electrons on the nitrogen atoms.^[^ ^28^ ^]^

## 3 Conclusion

In conclusion, a (hexaaza)octacene derivative is stabilized as its *σ*‐dimer^[^ ^20a^ ^]^ (i.e., a bioctacenyl). **4** is surprisingly stable due to its steric shielding, which sharply attenuates its reactivity in solution; and entirely suppresses it in the solid state. **4** demonstrates that sufficient (steric) protection of *π*‐systems at their most reactive positions should allow to prepare soluble azanonacenes and azadecacenes. We plan to make the higher biazaacenyls and investigate their properties with respect to applications in organic electronics. In the future, we will also investigate the mechanism of this unexpected dimerization reaction which is obscure at the moment.

## 4 Experimental Section

CCDC 2116992 (**3‐**H_4_), 2116995 (**4‐**H_4_), 2116993 (**4**), 2116994 (**5**) contains the supplementary crystallographic data for this paper. These data can be obtained free of charge from The Cambridge Crystallographic Data Centre via www.ccdc.cam.ac.uk/data_request/cif

Data related to this article are available via heiDATA, the institutional research data repository of Heidelberg University, under the following DOI: 10.11588/data/FABJEI.

### Procedure for the oxidation of **3‐**H_2_ to **4** and **5**

**3‐**H_2_ (84.0 mg, 72.4 µmol, 1.00 eq.) was dissolved in chloroform (300 mL) and heated to 60 °C. Manganese dioxide (252 mg, 2.90 mmol, 40.0 eq) was added and the reaction mixture was stirred for 1 h at 60 °C. The mixture was cooled down to r.t., filtered over Celite; the solvent was removed under reduced pressure. After flash column chromatography using PE/DCM (8:2 → 5:5), a mixture of **4‐**H_4_, **4** and pure **5** were isolated. The mixture of **4‐**H_4_ and **4** was dissolved in DCM (10 mL) and manganese dioxide (252 mg, 2.90 mmol, 40.0 eq) was added. The mixture was stirred at room temperature for 20 min and then filtered over Celite. Removal of the solvent gave pure **4**.

**4**: Habitus: brown solid. Melting point (mp) > 300 °C. Yield: 24% (20.2 mg, 8.69 µmol). *R*_f_ (PE:DCM 6:4) = 0.4.^1^H NMR (300 MHz, CD_2_Cl_2_, 295 K): *δ* [ppm]  = 9.90–9.60 (s, very broad, 2H), 8.56–8.53 (m, 2H), 8.26–8.23 (m, 2H), 8.03–8.00 (m, 2H), 7.87–7.84 (m, 2H), 7.71–7.63 (m, 4H), 7.46–7.34 (m, 4H), 1.50–1.47 (m, 84H), 0.79–0.68 (m, 84H). ^13^C{^1^H} NMR: Measurement not possible, most likely due to the diradical character of the compound. ATR‐IR: $\tilde{\nu }$  [cm^1^]  = 3065, 2941. 2889, 2863, 2725, 2137, 1737, 1588, 1524, 1461, 1386, 1310, 1223, 1161, 1137, 1101, 1064, 1032, 996, 920, 882, 754, 730, 676. HR‐MS (MALDI pos.) m/z: [M]^+^: calcd. for [C_144_H_186_N_12_Si_8_]^+^: 2307.3072; found: 2307.3024.

**5**: Habitus: orange‐brown solid. Mp > 300 °C. Yield: 53% (44.5 mg, 19.2 µmol). *R*_f_ (PE:DCM 6:4) = 0.75. ^1^H NMR (700 MHz, CDCl_3_, 295 K): *δ* [ppm]  =  8.53–8.52 (m, 4H), 8.14–8.12 (m, 4H), 7.77–7.75 (m, 4H), 7.54–7.52 (m, 4H), 6.00 (s, 4H), 1.36–1.33 (m, 168H). ^13^C{^1^H} NMR (176 MHz, CDCl_3_, 295 K): *δ* [ppm]  =  155.5, 153.8, 144.8, 143.1, 141.3, 139.9, 134.5, 131.7, 130.4, 128.1, 127.6, 123.3, 121.2, 111.4, 107.7, 102.3, 102.1, 55.3, 19.2, 19.2, 19.1, 19.1, 11.9, 11.7. ATR‐IR: $\tilde{\nu }$ [cm^1^]  =  2941, 2890, 2863, 1460, 1384, 1321, 1314, 1244, 1232, 1178, 1156, 1137, 1038, 1015, 995, 881, 756, 743, 731, 693, 674, 661, 574, 486, 466. HR‐MS (MALDI pos.) m/z: [M+2H]^+^: calcd. for [C_144_H_190_N_12_Si_8_]^+^: 2311.3385; found: 2311.3418.

## Conflict of Interest

The authors declare no conflict of interest.

## Supporting information

Supporting information

Supporting information

## Data Availability

The data that support the findings of this study are openly available at https://doi.org/10.11588/data/FABJEI and https://heidata.uni-heidelberg.de/.
