# Supplementary material for: A Stable Hexaazaoctacene Cruciform σ‐Dimer
Source: Adv Sci (Weinh). 2022 Jul 27;9(27):2202710. doi: 10.1002/advs.202202710 (PMC9507379; doi:10.1002/advs.202202710)
Supplement: Supplementary file 1 — Supporting information [file ADVS-9-2202710-s001.pdf]

ADVANCED SCIENCE
Open Access

Supporting Information

for *Adv. Sci.*, DOI 10.1002/advs.202202710

A Stable Hexaazaoctacene Cruciform σ-Dimer

Steffen Maier, Fabian Jester, Marvin T. Hoffmann, Frank Rominger, Jan Freudenberg, Andreas Dreuw* and Uwe H. F. Bunz**

Supporting Information
©Wiley-VCH 2016
69451 Weinheim, Germany

A Stable Hexaazaoctacene Cruciform σ-Dimer

Steffen Maier, Fabian Jester, Marvin T. Hoffmann, Frank Rominger, Jan Freudenberg*, Andreas Dreuw*, Uwe H. F. Bunz*

Table of Contents

1 Experimental Procedures ... 3
1.1 Materials and Methods ... 3
1.2 Syntheses ... 3
2. Results and Discussion ... 6
2.2. UV-vis Spectra ... 6
2.3 Calculations ... 8
2.3.1 NICS Calculations ... 8
2.3.2 AICD Calculations ... 8
2.3.3 Calculated Bond Lengths ... 9
2.3.4 FMO calculations ... 10
2.3.5 Excited Electronic States Calculations ... 12
2.3.6 Coordinates of the Optimized Geometries ... 13
2.4 Comparison of 4 with 4-H_4 and 3-H_2 ... 23
2.5 NMR Spectroscopy ... 25
2.6 UV/vis Stability Studies ... 30
2.7 Cyclic Voltammetry ... 31
2.8 EPR Spectroscopy ... 32
2.9 Crystallogrpahic Data ... 32
2.9 References ... 38

1 Experimental Procedures

1.1 Materials and Methods

Column chromatography was performed using silica gel obtained from Sigma Aldrich (particle size: 0.032−0.062 mm). Preparative gel permeation chromatography (GPC) was performed on Bio-Beads® (S-X1 Beads, 200 – 400 Mesh, crosslinked polystyrene) purchased from Bio-Rad Laboratories, Inc., using toluene as eluent. High performance liquid chromatography was performed on a Jasco 250 system using a Reprosil Pur 120 Si 5 µm 250 x 20 mm column obtained from Dr. Maisch. We provide further details about the used manganese(IV)oxide because we noticed that an old batch was not able to oxidize **4-H$_4$** to **4**.[1] Manganese(IV)oxide was purchased from Sigma Aldrich (Article ID: 8.05958.1000, Lot: S7610158028) bearing the following specification “precipitated active for synthesis”. Certificate of Analysis: Assay (idodometric, calc. on dried substance): 90.5%; Loss on drying: 3.1%. RUPHOS Pd G1 is an abbreviation for (2-Dicyclohexylphosphino-2′,6′-diisopropyl-1,1′-biphenyl)[2-(2-aminoethyl)-phenyl)]-palladium(II). NMR spectra were recorded on Bruker Avance Spectrometers (Bruker Avance III 300, Bruker Avance III 400, Bruker Avance III 600, Bruker Avance Neo, using the specified frequency. Chemical shifts (*δ*) are given in parts per million (ppm) relative to internal solvent signals.[S1] The following abbreviations describe the signal multiplicities: s = singlet, d = doublet, dd = doublet of doublets, m = multiplet. High-resolution mass spectra (HRMS) were obtained by (matrix-assisted) laser desorption/ionization (LDI/MALDI) using *trans*-2-[3-(4-*tert*-butylphenyl)-2-methyl-2-propenylidene]malononitrile (DCTB) as matrix, electrospray ionisation (ESI) or direct analysis in real time (DART) experiments. Infrared (IR) spectra were recorded as neat oils or powders of the respective analytes using a Jasco FT/IR-4100 spectrometer. Cyclovoltammetry (CV) measurements were performed on a Metrohm Autolab PGSTAT101. Absorption spectra were recorded on a Jasco UV-Vis V-660 or Jasco UV-Vis V 670. Fluorescence spectra were recorded on a Jasco FP-6500. Melting points (m.p.) were determined in open glass capillaries with a Melting Point Apparatus MELTEMP (Electrothermal, Rochford, UK). All calculations were performed using Gaussian16.[S2] TMS groups were used instead of TIPS groups to simplify calculations. First, the gas‐phase ground‐state equilibrium geometry of the molecules was optimized at the B3LYP/def2‐SVP level of theory. FMO calculations were performed starting from the optimized geometries on the B3LYP/def2-TZVP level of theory. For some molecules another approach was used, which is described in the section calculation. 1,4-Bis((triisopropylsilyl)ethynyl)phenazine-2,3-diamine (**S1**)[S3], 4,5-Dibromobenzene-1,2-diol (**S2**)[S4], 1,4-Bis((triisopropylsilyl)ethynyl)naphthalene-2,3-diamine (**2**)[S5] were synthesized according to literature procedures.

1.2 Syntheses

2,3-Dibromo-6,13-bis((triisopropylsilyl)ethynyl)-5,14-dihydroquinoxalino[2,3-b]phenazine / 9,10-dibromo-6,13-bis((triisopropylsilyl)ethynyl)-5,14-dihydroquinoxalino[2,3-b]phenazine, mixture of tautomers (1)

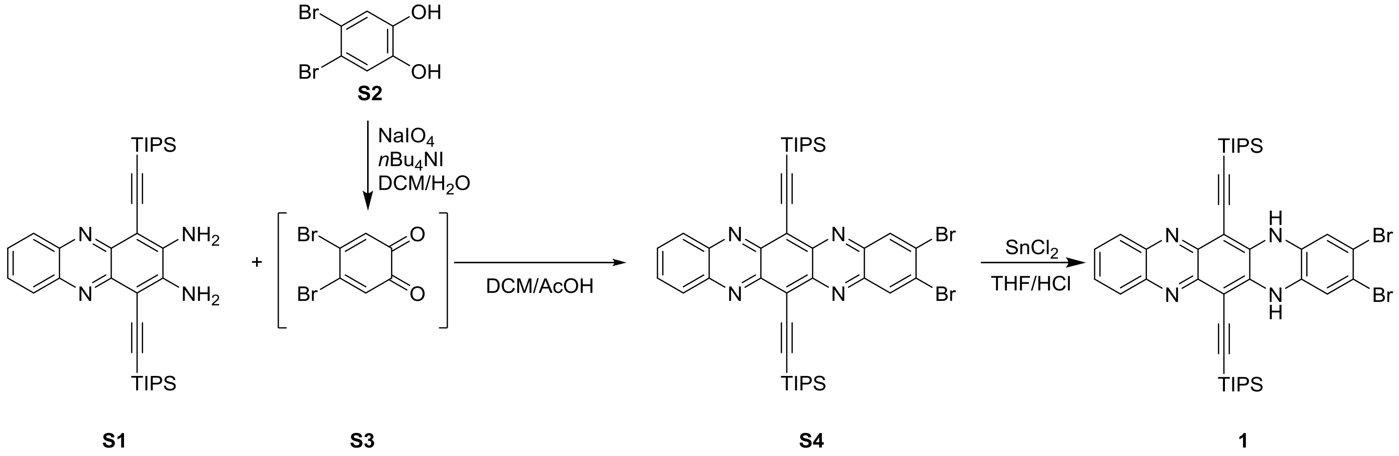

Diol **S2** (5.27 g, 19.7 mmol, 1.00 eq.) was dissolved in 100 mL of DCM. Subsequently sodium meta periodate (5.05 g, 23.6 mmol, 1.20 eq.) and NBu$_4$I (243 mg, 787 µmol, 4 mol-%.) were added. After addition of 100 mL of deionized water, the reaction mixture was stirred for 1 h at room temperature. The reaction mixture was extracted

[1] When using **3** as starting material, we observed the formation of a product with a plausible NMR spectrum but a m/z of 1270, which indicates the addition of a CCl_3 group. This product shows no vibronic finger structure.

with DCM and the combined organic layers were dried over $MgSO_4$. After removal of the solvent in vacuo, crude quinone **S3** was dissolved in a mixture of 40 mL DCM and 40 mL acetic acid. After addition of diamine **S1** (5.00 g, 8.76 mmol, 0.44 eq) the reaction mixture was stirred at room temperature overnight. The end of the reaction was monitored by TLC. After completion, an aqueous solution of $NaHCO_3$ was added to neutralize the solution. The aqueous phase was extracted with DCM, the combined organic layers were dried over $MgSO_4$ and the solvent was removed under reduced pressure. The crude product was purified by column chromatography on silica gel (PE:DCM 8:2, 7:3) to yield dibromotetraazapentacene **S4** as dark green solid (4.72 g, 5.91 mmol, 67%, R_f(PE:DCM 7:3)=0.4)), which was used without further characterization. **S4** (4.72 g, 5.89 mmol, 1.00 eq) was dissolved in 200 mL of THF. A solution of $SnCl_2$ (13.3 g, 58.9 mmol, 10.0 eq.) in 200 mL of 6 M HCl was added dropwise. The reaction mixture was stirred at room temperature for 4 h and extracted with DCM. The combined organic layers were washed three times with a 2 M NaOH solution and dried over $MgSO_4$. After removal of the solvent the product was yielded as a mixture of tautomers (ratio determined by ^{1}H NMR: 4:1) as a violet-golden solid (3.50 g, 4.37 mmol, 74%).

Tautomer A:

^{1}H NMR ($CDCl_3$), 600 MHz, 295 K): δ [ppm] = 8.16 (s, 2 H), 7.20 (s, 2H), 6.82-6.79 (m, 2H), 6.52-6.49 (m, 2H), 1.25-1.24 (m, 42 H).

^{13}C{^{1}H} NMR (151 MHz, $CDCl_3$, 295 K): δ [ppm] = 144.6, 141.4, 140.4, 132.8, 128.0, 124.1, 123.9, 114.2, 105.1, 99.1, 96.7, 19.0, 11.5.

Tautomer B:

^{1}H NMR ($CDCl_3$) 600 MHz, 295 K): δ [ppm] = 7.93-7.92 (m, 2H), 7.58-7.56 (m, 2H), 6.98 (s, 2H), 6.65 (s, 2H), 1.25-1.24 (m, 42 H).

^{13}C{^{1}H} NMR (151 MHz, $CDCl_3$, 295 K): δ [ppm] = 143.3, 142.4, 138.2, 129.2, 128.9, 128.8, 117.8, 117.1, 105.7, 99.1, 98.6, 19.0, 11.5.

Tautomer A+B

ATR-IR: $\tilde{\nu}$ [cm^{-1}] = 3372, 2939, 2888, 2862, 2161, 2153, 2144, 2139, 2122, 2097, 1611, 1599, 1574, 1523, 1494, 1453, 1415, 1398, 1387, 1366, 1328, 1308, 1287, 1269, 1253, 1236, 1224, 1202, 1154, 1125, 1109, 1080, 1038, 1025, 1014, 993, 950, 918, 907, 880, 870, 842, 817, 809, 751, 730, 704, 669, 659, 645, 623, 587, 581, 552, 547, 534, 517, 503, 492, 485, 478, 455, 443, 430, 425, 420, 418, 410, 404.

HR-MS (MALDI pos.) m/z: $[M]^+$: calcd. for $[C_{40}H_{50}N_4Si_2{}^{79}Br_2]^+$: 800.1935; found: 800.1940.

Melting point: > 300 °C.

***R*$_f$** (PE:DCM 7:3) = 0.35.

6,10,15,19-Tetrakis{[tri(propan-2-yl)silyl]ethynyl}-7,18-dihydrobenzo[*b*]-quinoxalino[2',3':6,7]quinoxalino[2,3-*i*]phenazine (3-H$_2$)

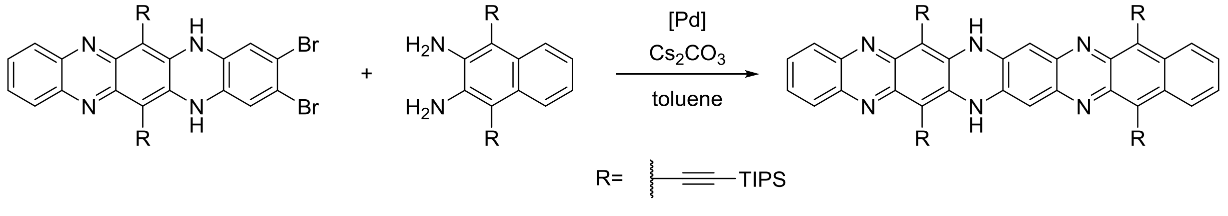

1 **2** **3-H$_2$**

1 (300 mg, 374. µmol, 1.10 eq., mixture of tautomers), **2** (176 mg, 340 µmol, 1.00 eq.) was dissolved in 50 mL of dry toluene under an argon atmosphere. The resulting solution was degassed for 30 min. Caesium carbonate (553 mg, 1.70 mmol, 5.00 eq.) and RuPhos Pd G1 (27.8 mg, 34.0 µmol, 10.0 mol%.) were added and the reaction mixture was stirred for 18 h at 140 °C. After cooling to room temperature, a saturated ammonium chloride solution was added and the mixture was extracted with DCM. The combined organic layers were dried over $MgSO_4$ and the crude product was purified by flash column chromatography PE/DCM 8:2 → 5:5 to yield the

product (also a fraction of **3-H$_4$** formed, which oxidized rapidly under ambient conditions to **3-H$_2$**) as a violet solid (209 mg, 53%).

^{1}H NMR (600 MHz, CDCl$_3$, 295 K): δ [ppm] = 8.67 – 8.64 (m, 2H), 8.09 (s, 2H), 8.08 – 8.06 (m, 2H), 7.70 – 7.68 (m, 2H), 7.61 – 7.58 (m, 2H), 7.12 (s, 2H), 1.35 – 1.31 (m, 84H).

^{13}C{^{1}H} NMR (151 MHz, CDCl$_3$, 295 K): δ [ppm] = 144.9, 142.8, 141.9, 141.4, 135.4, 134.6, 134.1, 129.6, 129.6, 127.6, 127.5, 119.7, 107.3, 107.2, 106.8, 103.3, 101.2, 98.9, 19.2, 19.1, 11.8, 11.6.

ATR-IR: $\tilde{\nu}$ [cm^{-1}] = 3358, 2941, 2863, 1586, 1438, 1389, 1220, 1092, 1016, 880, 756, 727, 676, 661, 653, 572.

HR-MS (MALDI pos.) m/z: [M+2H]$^+$: calcd. for [$C_{72}H_{98}N_6Si_4$]$^+$: 1158.6925; found: 1158.6920.

Melting point: > 300 °C.

***R*$_f$** (PE:DCM 4:6) = 0.4.

6,6',10,10',15,15',19,19'-Octakis{[tri(propan-2-yl)silyl]ethynyl}-7,7',18,18'-tetrahydro-8,8'-bibenzo[*b*]quinoxalino[2',3':6,7]quinoxalino[2,3-*i*]phenazine (4-H$_4$)

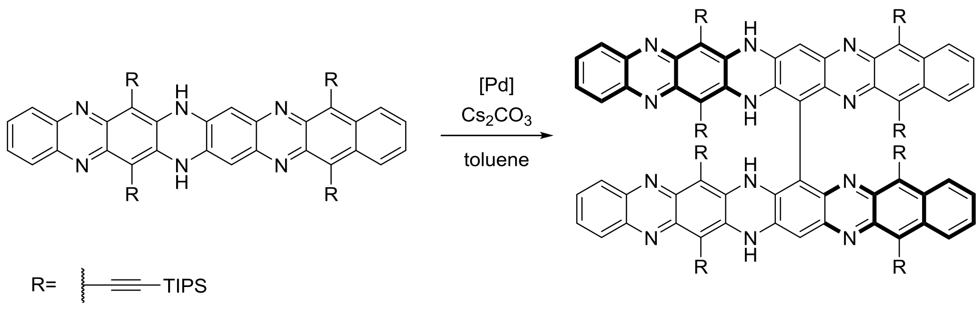

3-H$_2$ **4-H$_4$**

3-H$_2$ (10.0 mg, 8.64 µmol, 1.00 eq.) was dissolved in dry toluene (15 mL). Subsequently, the reaction mixture was degassed, caesium carbonate (14.1 mg, 43.2 µmol, 5.00 eq.) and RuPhos Pd G1 (1.41 mg, 1.73 µmol, 20 mol%.) was added and stirred for 20 h at 140 °C. The mixture was allowed to cool to rt and was neutralized with aqueous sodium hydrogen carbonate solution. The phases were separated and the aqueous layer was extracted with DCM. The combined organic phases were dried over magnesium sulfate and the solvent was removed under reduced pressure. After flash column chromatography (DCM), preparative high-performance-liquid chromatography (PE/DCM 9:1) and gel permeation chromatography (toluene), the purified product was isolated as a violet solid (4.00 mg, 40%).

^{1}H NMR (700 MHz, CDCl$_3$, 295 K): δ [ppm] = 8.61 (d, *J* = 8.7 Hz, 2H), 8.49 (d, *J* = 8.8 Hz, 2H), 8.16 (s, 2H), 8.01 (d, *J* = 8.0 Hz, 2H), 7.89 (d, *J* = 8.9 Hz, 2H), 7.70 (s, 2H), 7.64 – 7.61 (m, 2H), 7.60 – 7.57 (m, 2H), 7.51 – 7.49 (m, 2H), 7.45 – 7.42 (m, 2H), 7.30 (s, 2H), 1.40 – 1.37 (m, 84H), 1.00 – 0.95 (m, 42H), 0.90 – 0.86 (m, 42H).

^{13}C{^{1}H} NMR (176 MHz, CDCl$_3$, 295 K): δ [ppm] = 144.5, 143.1, 142.8, 142.7, 142.5, 141.9, 141.6, 140.7, 135.3, 135.2, 134.3, 134.0, 133.6, 133.4, 129.5, 129.4, 129.4, 129.4, 127.6, 127.5, 127.3, 127.2, 120.2, 119.8, 109.2, 108.7, 108.2, 106.8, 106.1, 106.0, 103.5, 102.8, 102.1, 100.8, 98.9, 97.6, 19.2, 19.2, 18.9, 18.8, 18.8, 11.8, 11.7, 11.6, 11.5.

ATR-IR: $\tilde{\nu}$ [cm^{-1}] = 3349, 2923, 2861, 2136, 1721, 1581, 1469, 1426, 1267, 1205, 1099, 1032, 1018, 880, 756, 728, 675, 660, 580, 482.

HR-MS (MALDI pos.) m/z: [M]$^+$: calcd. for [$C_{144}H_{190}N_{12}Si_8$]$^+$: 2311.3385; found: 2311.3531.

Melting point: > 300 °C.

***R*$_f$** (PE:DCM 6:4) = 0.35

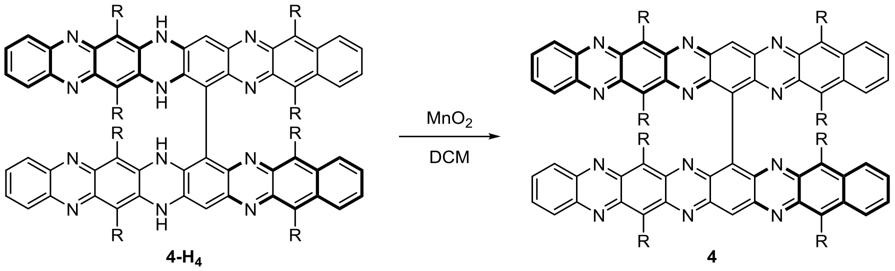

4-H$_4$ (50.0 mg, 21.6 µmol, 1.00 eq.) was dissolved in DCM (20 mL). Manganese dioxide (75.1 mg, 864 µmol, 40.0 eq.) was added and the reaction mixture was stirred for 1 h at r.t.. The reaction mixture was filtered over Celite®. After removing of the solvent pure **4** was isolated.

Analytical data is shown in the main text.

2. Results and Discussion

2.2. UV-vis Spectra

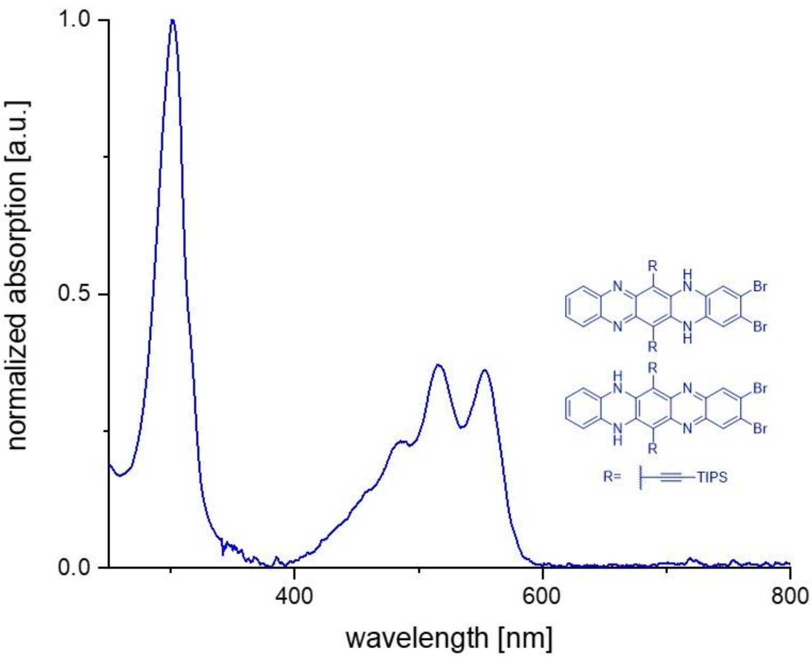

Figure S1. Normalized absorption spectrum of **1** in DCM.

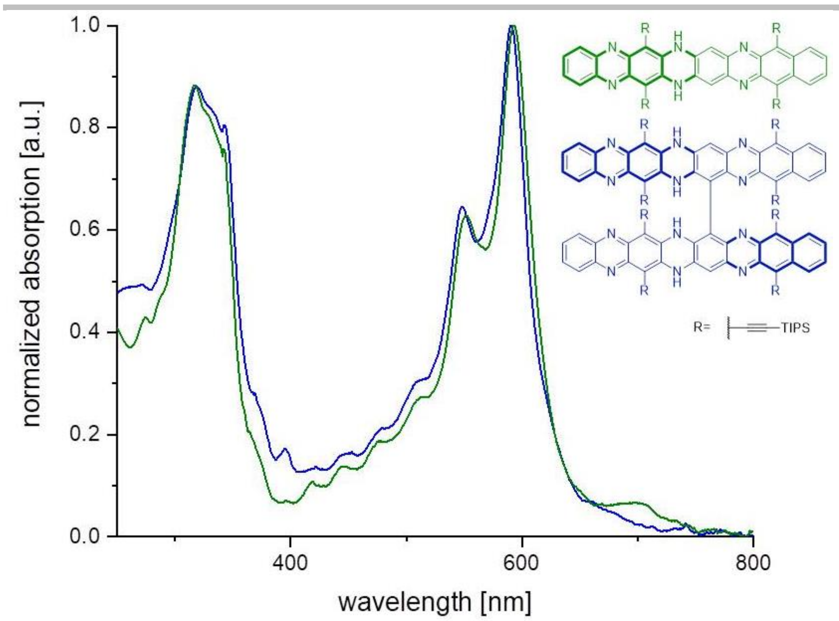

Figure S2. Normalized absorption spectra of **3-H$_2$** (green) and **4-H$_4$** (blue).

2.3 Calculations

2.3.1 NICS Calculations

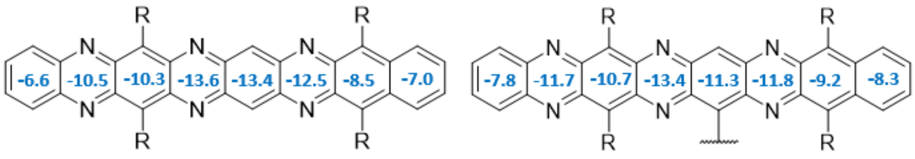

Figure S3. NICS(1) values[S6] of monomer **3** (left) and its dimer **4** (right, Gaussian 16 B3LYP/def2-SVP; TMS groups were used instead of TIPS groups to reduce the computational cost).

Compared to hypothetical **3**, σ-dimerization to **4** does slightly decrease aromaticity at the rings involved in dimer formation from -13.4 ppm to -11.3 and at those in the vicinity, whereas the most outer rings slightly gain in aromaticity.

2.3.2 AICD Calculations

ACID-plots were calculated starting from the optimized geometries using AICD-3.0.3 [S7] using the CSGT-method at the B3LYP/def2-TZVP IOP(10/93=1) level of theory.

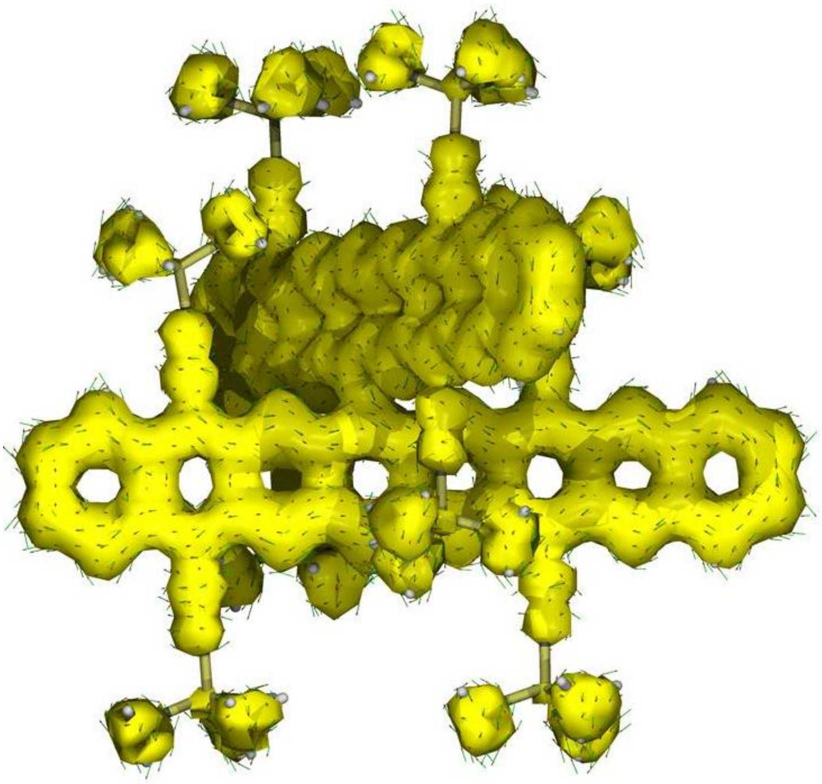

Figure S4. AICD plot of **4** (isovalue = 0.02; magnetic field vector is orientated out of plane; CSGT-method B3LYP/def2-TZVP IOP(10/93=1; TMS groups were used instead of TIPS groups to reduce the computational cost).

2.3.3 Calculated Bond Lengths

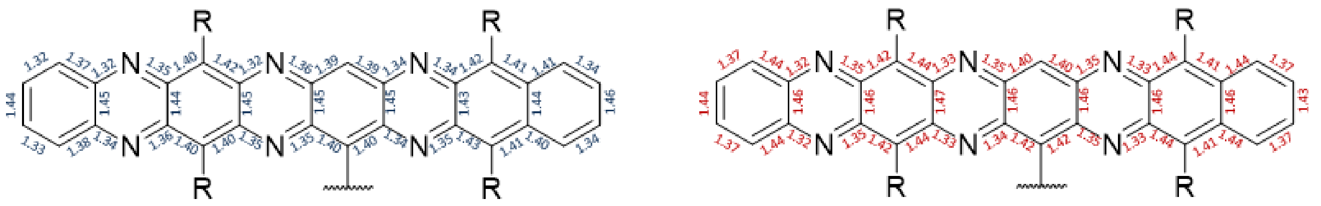

Figure S5. Calculated (red) and measured (blue) bond lengths of the dimer **4** (TMS groups were used instead of TIPS groups to reduce the computational cost).

2.3.4 FMO calculations

3 and **3-H$_2$**: Calculations were performed using Gaussian16. TMS groups were used instead of TIPS groups to simplify calculations. First, the gas-phase ground-state equilibrium geometry of the molecules was optimized at the B3LYP/def2-SVP level of theory. Afterwards, the received geometries were refined using the B3LYP/def2-TZVP level of theory. FMO calculations were performed starting from the optimized geometries on the B3LYP/def2-TZVP level of theory.

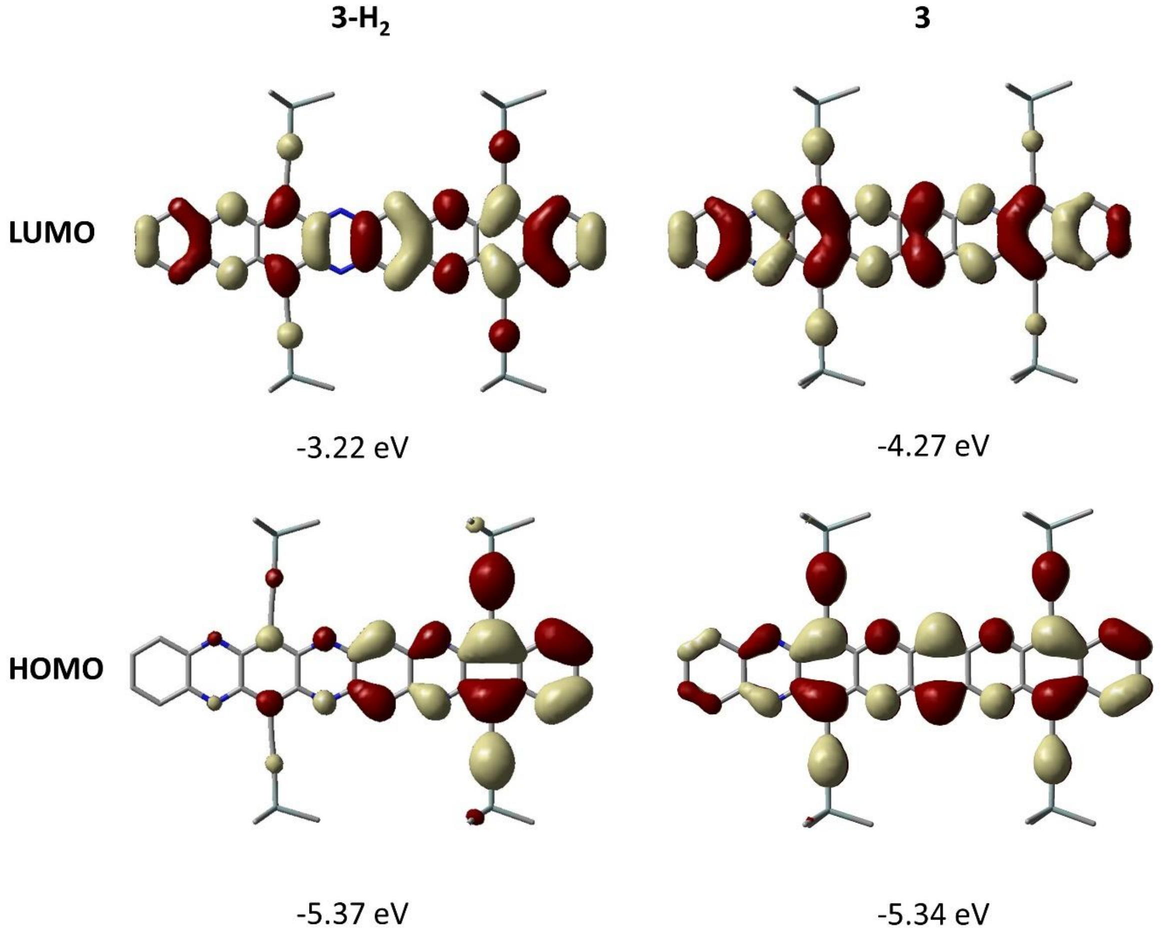

Figure S6. Calculated FMOs of **3-H$_2$** (left) and **3** (right) with their energies.

4 and **4-H$_4$**: Frontier molecular orbital (FMO) calculations were performed in two different ways because the bulky TIPS groups are important for the geometry imparted upon the molecule but also need an extremely high computational cost.

Method A: First, the gas-phase ground-state equilibrium geometry of the molecules was optimized at the B3LYP/def2-SVP level of theory and a single point calculation provided the resulting FMOs and their energies.

Method B: A single point energy calculation on the B3LYP/def2-TZVP level of theory was performed using the geometries obtained by crystal structure.

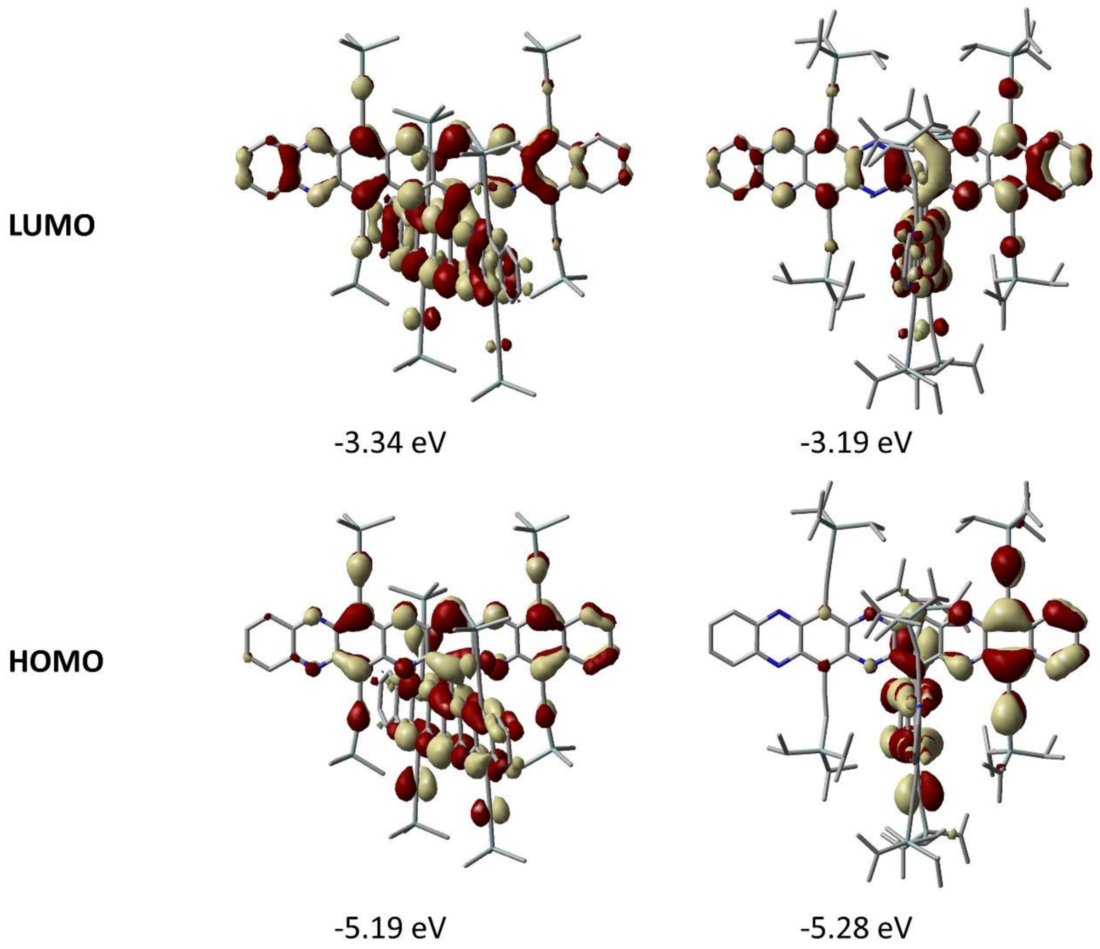

Figure S7. Calculated FMOs of **4-H$_4$** using method A (left, torsion angle 65.8°) and method B (right, torsion angle 84.5°).

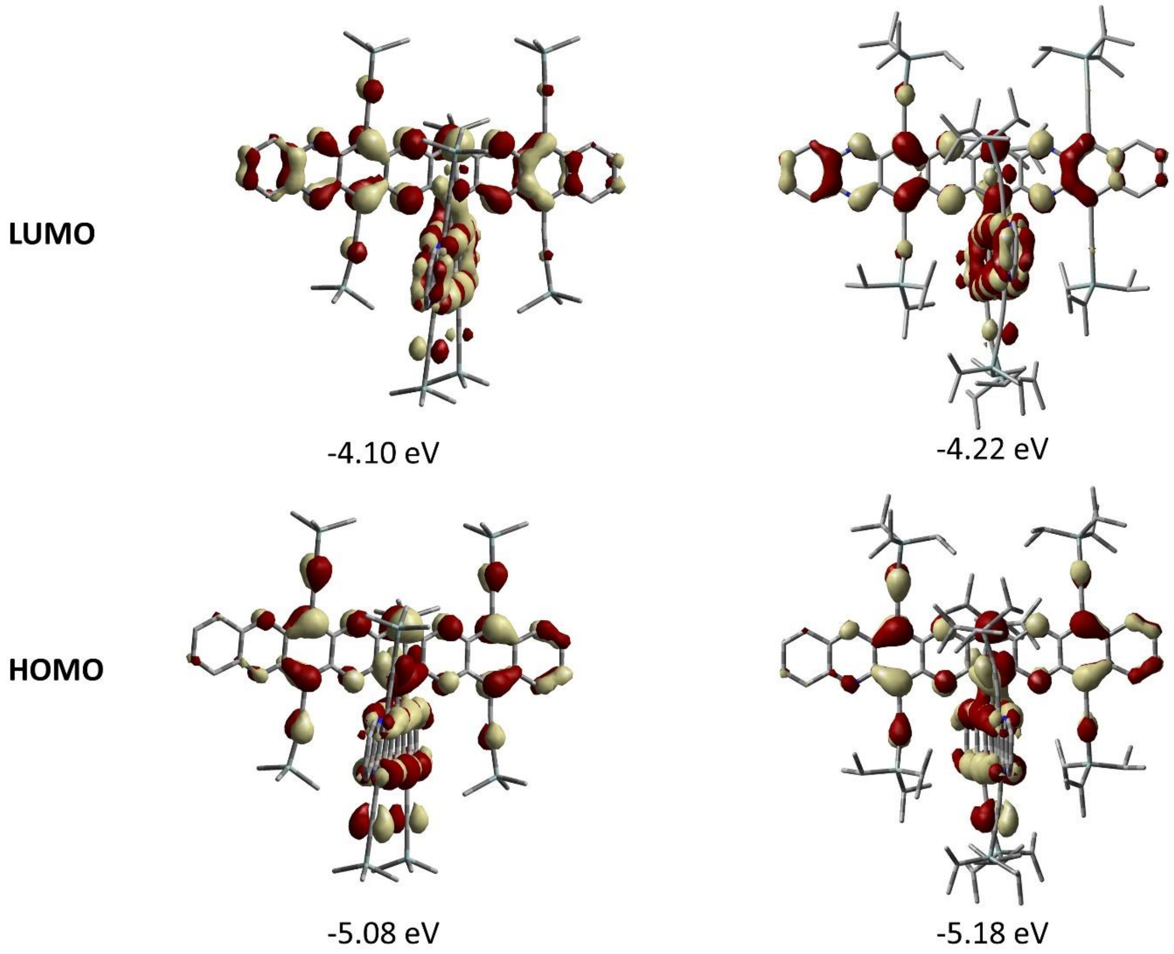

Figure S8. Calculated FMOs of **4** under use of way a) (left, torsion angle 68.5°) and way b (right, 85.3°).

2.3.5 Excited Electronic States Calculations

The TDDFT calculations were performed on the CAM-B3LYP/pcseg-1 level of theory as implemented in the Q-Chem software package[S8] for the same structure used in the FMO calculations for comparability (Method A). Furthermore, a C-PCM model was used to describe the influence of the solvent used in the experiments, namely DCM. The first 40 excited singlet states were calculated.

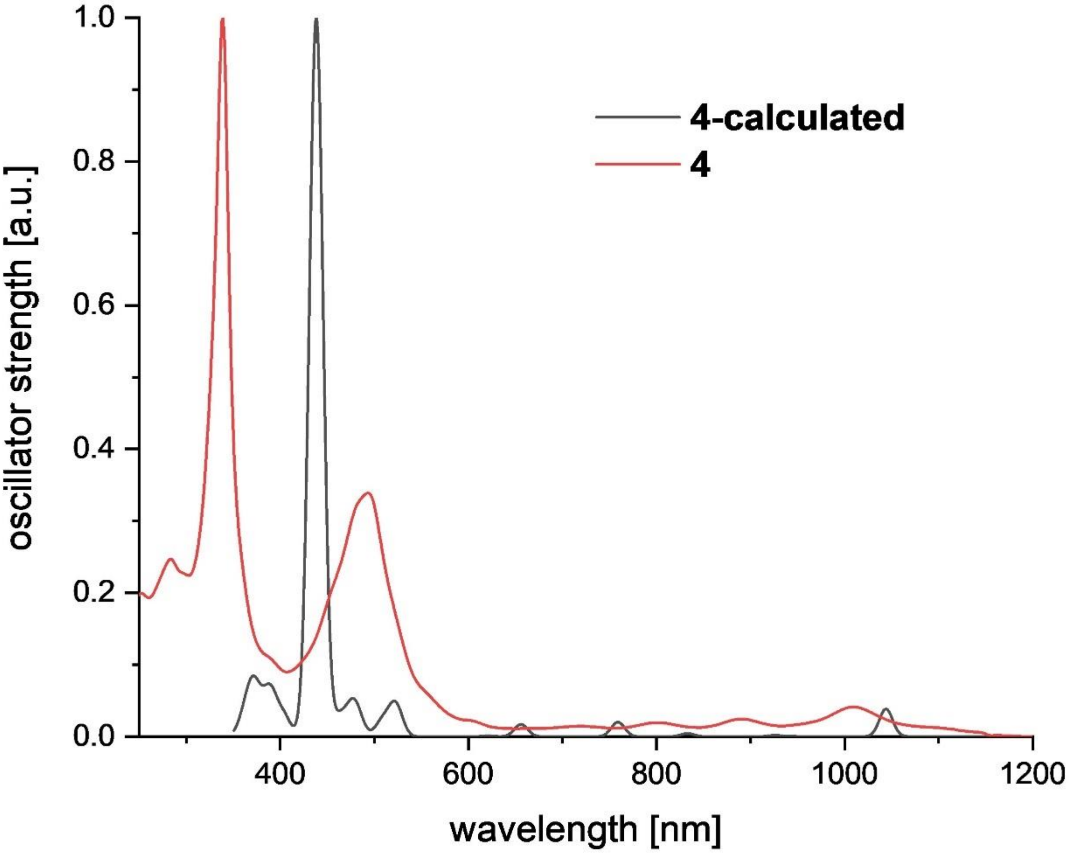

Figure S9. Calculated (black) UV/VIS spectrum of the dimer **4** (CAM-B3LYP/pcseg-1/PCM(DCM) in comparison to the experimentally (red) measured UV/VIS-spectra.

2.3.6 Coordinates of the Optimized Geometries

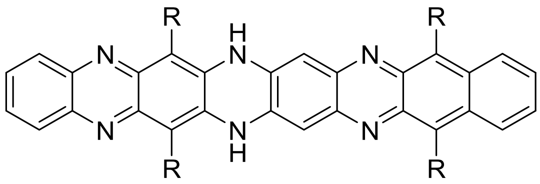

C	-10.16294300	0.70962400	0.00003300
C	-10.16294000	-0.70967000	0.00004700
C	-8.98796400	-1.40831200	0.00003800
C	-7.74843700	-0.71670900	0.00001500
C	-7.74844000	0.71667400	0.00000100
C	-8.98797000	1.40827200	0.00001100
N	-6.59685400	-1.40757000	0.00000600
C	-5.46347200	-0.72186400	-0.00001600
C	-5.46347500	0.72183900	-0.00002800
N	-6.59686000	1.40754000	-0.00002100
C	-4.21067400	-1.43183200	-0.00002700
C	-3.02464800	-0.71553100	-0.00005100
C	-3.02465100	0.71551700	-0.00006200
C	-4.21068000	1.43181300	-0.00005200
N	-1.80803000	-1.35248800	-0.00006800
C	-0.57981900	-0.72314300	-0.00006400

C	-0.57982200	0.72313900	-0.00007100
N	-1.80803600	1.35247900	-0.00008400
C	0.59742700	-1.41331500	-0.00005400
C	1.84270300	-0.72513200	-0.00005000
C	1.84270000	0.72514000	-0.00005600
C	0.59742000	1.41331600	-0.00006600
C	-4.16355100	2.84550600	-0.00007700
C	-4.04183700	4.05467200	-0.00008100
C	-4.16353900	-2.84552500	-0.00001400
C	-4.04181600	-4.05469000	-0.00000700
N	2.97361300	-1.40897600	-0.00003800
C	4.13178600	-0.72050800	-0.00003300
C	4.13178300	0.72052600	-0.00004000
N	2.97360700	1.40898900	-0.00005100
C	5.35929800	-1.43244500	-0.00001800
C	6.57944900	-0.71962800	-0.00001600
C	6.57944600	0.71965700	-0.00002500
C	5.35929100	1.43246800	-0.00003400
C	7.83261800	-1.39744800	-0.00000700
C	9.00954000	-0.70895100	-0.00000700
C	9.00953700	0.70899200	-0.00001500
C	7.83261100	1.39748300	-0.00002400
C	5.34584800	-2.84861600	-0.00000700
C	5.36272300	-4.06299000	0.00001100
C	5.34583500	2.84864000	-0.00003800
C	5.36270700	4.06301400	-0.00003400
Si	-3.98405100	-5.89699000	-0.00002600
C	-4.85540600	-6.51331700	-1.54421100
C	-4.85505400	-6.51334200	1.54434800
C	-2.18240800	-6.43197800	-0.00023400
Si	-3.98412400	5.89697300	0.00007100
C	-4.85703400	6.51319300	1.54341900
C	-4.85360700	6.51338500	-1.54513700
C	-2.18249700	6.43201200	0.00208000
Si	5.33108800	-5.89933400	0.00008400
C	4.42880800	-6.48158200	-1.54216900
C	4.42978700	-6.48148100	1.54294700
C	7.10052500	-6.53345800	-0.00045500
Si	5.33106900	5.89935700	-0.00000200
C	4.42906400	6.48157700	-1.54242700
C	7.10050500	6.53348300	-0.00023500
C	4.42948700	6.48153100	1.54268700
H	-11.10749800	1.23882200	0.00004100
H	-11.10749200	-1.23887200	0.00006400
H	-8.96413900	-2.49018200	0.00004800
H	-8.96415000	2.49014100	0.00000100
H	-1.83483800	-2.36346300	-0.00005600
H	-1.83484800	2.36345400	-0.00008200
H	0.61395900	-2.49568200	-0.00004800
H	0.61394800	2.49568400	-0.00006600
H	7.82769800	-2.47844300	0.00000000
H	9.95028500	-1.24491300	0.00000000
H	9.95027900	1.24495800	-0.00001600
H	7.82768700	2.47847800	-0.00003100
H	-4.36307200	-6.14951300	-2.44828800
H	-4.85681600	-7.60597200	-1.57767600
H	-5.89270800	-6.17377600	-1.56983800
H	-4.36251700	-6.14954800	2.44831900
H	-4.85645000	-7.60599700	1.57779800
H	-5.89235100	-6.17380700	1.57021500
H	-2.10498300	-7.52225700	-0.00026300

H	-1.65764500	-6.06282300	-0.88412600
H	-1.65745300	-6.06285900	0.88355900
H	-4.36556200	6.14938800	2.44796500
H	-4.85854900	7.60584600	1.57692000
H	-5.89433600	6.17357900	1.56801700
H	-4.36018700	6.14961900	-2.44863800
H	-4.85495900	7.60604100	-1.57855100
H	-5.89088300	6.17386400	-1.57203500
H	-1.65856300	6.06272300	0.88640600
H	-1.65669400	6.06305700	-0.88127800
H	-2.10510400	7.52229300	0.00235900
H	4.93499900	-6.13981900	-2.44727200
H	4.37972900	-7.57324000	-1.57503300
H	3.40733100	-6.09671200	-1.56667600
H	4.93653200	-6.13962900	2.44770600
H	4.38076000	-7.57313700	1.57593200
H	3.40831400	-6.09664000	1.56806200
H	7.12121000	-7.62641700	-0.00046200
H	7.64305200	-6.18881300	-0.88336000
H	7.64358600	-6.18881700	0.88212500
H	3.40762500	6.09662300	-1.56715400
H	4.37990000	7.57323200	-1.57526800
H	4.93546700	6.13988300	-2.44743700
H	7.64338100	6.18894900	0.88250000
H	7.12118700	7.62644300	-0.00036800
H	7.64321900	6.18873600	-0.88298600
H	4.38053500	7.57319000	1.57569100
H	3.40798000	6.09676600	1.56757900
H	4.93601900	6.13961500	2.44754100

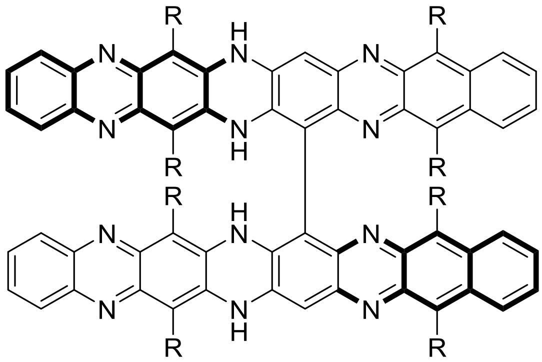

C	-7.94541800	5.03789300	5.57384800
C	-6.81380000	5.91566700	5.64483500
C	-5.63554200	5.59626300	5.02261100
C	-5.51787500	4.37062500	4.28664700
C	-6.66429900	3.48103100	4.21550000
C	-7.87748300	3.85703300	4.88223400
N	-4.37345300	4.06227500	3.68174000
C	-4.29871200	2.90717200	3.00076800
C	-5.44634500	2.01429800	2.93387300
N	-6.59869700	2.33321500	3.54580600
C	-3.07292400	2.56608700	2.33942400
C	-2.99904100	1.34693700	1.61737700
C	-4.13931400	0.44415300	1.57341900
C	-5.35836300	0.77829800	2.21832700
N	-1.85504200	1.03143900	0.96358100
C	-1.77967900	-0.11984200	0.31784100
C	-2.91483400	-1.04362800	0.31145000
N	-4.05059300	-0.73362500	0.91253200
C	-0.57778900	-0.46835900	-0.40515800
C	-0.50789000	-1.69131600	-1.05742500

C	-1.63591800	-2.61446800	-1.03334400
C	-2.79253900	-2.28913300	-0.37110800
N	0.60309200	-2.09139900	-1.76041400
C	0.74509500	-3.29878800	-2.42141400
C	-0.35929000	-4.20004000	-2.39479600
N	-1.48785500	-3.80748200	-1.70314900
C	1.92103000	-3.63888400	-3.09863900
C	2.01032400	-4.91000400	-3.77983900
C	0.89836000	-5.81035800	-3.75317100
C	-0.29828100	-5.43760400	-3.04298200
C	3.18147900	-5.29643000	-4.48197100
C	3.25616300	-6.51418000	-5.13070400
C	2.15727800	-7.40139900	-5.10392500
C	1.00358300	-7.05382400	-4.42794200
C	-6.47279000	-0.09847900	2.16321300
C	-1.96415200	3.44648400	2.43618700
C	3.02379700	-2.73731300	-3.11167100
C	-1.43051700	-6.29791500	-2.97423600
C	-1.04315000	4.24159200	2.58191300
C	-7.43830100	-0.85124300	2.11875200
C	-2.43110700	-7.00188300	-2.88113200
C	3.99973400	-1.99476100	-3.14669900
C	6.82213300	-5.89289300	5.65766500
C	7.95318500	-5.01468300	5.58282900
C	7.88402000	-3.83613900	4.88738800
C	6.66997600	-3.46314400	4.22049800
C	5.52409000	-4.35313900	4.29554700
C	5.64312400	-5.57628500	5.03542800
N	6.60310600	-2.31758400	3.54711100
C	5.44999100	-2.00116800	2.93539300
C	4.30297800	-2.89447000	3.00608600
N	4.37898300	-4.04742400	3.69058600
C	5.36092400	-0.76739100	2.21615100
C	4.14096000	-0.43549500	1.57169900
C	3.00138100	-1.33906000	1.61914300
C	3.07660100	-2.55620100	2.34445500
N	4.05064000	0.74051700	0.90779000
C	2.91406900	1.04797900	0.30688900
C	1.77990400	0.12298300	0.31630000
N	1.85672300	-1.02643600	0.96515400
C	2.78977200	2.29191200	-0.37832100
C	1.63249100	2.61408000	-1.04105500
C	0.50619300	1.68882400	-1.06340600
C	0.57741700	0.46810600	-0.40716600
N	1.48204800	3.80580400	-1.71270100
C	0.35281400	4.19467300	-2.40542300
C	-0.74922000	3.29051400	-2.43184700
N	-0.60492300	2.08472000	-1.76857700
C	0.28900400	5.43115000	-3.05544500
C	-0.90811000	5.79997900	-3.76692500
C	-2.01738700	4.89636700	-3.79396600
C	-1.92505800	3.62616900	-3.11144700
C	-1.01637300	7.04254700	-4.44281200
C	-2.17035400	7.38619700	-5.12029300
C	-3.26655200	6.49568900	-5.14753300
C	-3.18890900	5.27870400	-4.49771700
C	1.96881600	-3.43757800	2.44347400
C	6.47535900	0.10911600	2.15655300
C	1.41942100	6.29381800	-2.98759800
C	-3.02453700	2.72063100	-3.12658900
C	7.44060100	0.86189000	2.10674000

C	1.04898700	-4.23381800	2.59041100
C	-3.99748400	1.97433100	-3.16501300
C	2.41933400	6.99873500	-2.89465600
Si	0.23034600	5.51956900	2.97524400
C	0.79070100	5.26179000	4.76075600
C	-0.56331200	7.22227400	2.77451900
C	1.70520600	5.35268500	1.80654000
Si	-8.89975400	-1.97625700	2.07716200
C	-10.42044400	-0.97667900	1.56848100
C	-9.14857600	-2.70995900	3.80038700
C	-8.57353400	-3.35269800	0.82371400
Si	-3.91509900	-8.11033200	-2.76322700
C	-4.03618300	-8.76153300	-0.99607900
C	-3.68083600	-9.53009300	-3.98347500
C	-5.44934300	-7.10154400	-3.20190900
Si	5.58565300	-1.03605900	-3.31552700
C	6.88788100	-1.85683200	-2.22633200
C	5.32134200	0.75444400	-2.79336700
C	6.08921900	-1.12442000	-5.13476900
Si	8.89488900	1.99527100	2.04464000
C	10.29512000	1.24684700	3.06705000
C	8.38798500	3.67202200	2.75352600
C	9.43368400	2.18974400	0.24265800
Si	-0.22137800	-5.51533900	2.98263100
C	-1.70496800	-5.33872600	1.82662900
C	-0.76775700	-5.27308400	4.77467200
C	0.57106800	-7.21602700	2.76117600
Si	-5.57813000	1.00905000	-3.34441000
C	-5.32222100	-0.76950300	-2.77938700
C	-6.04681000	1.05803000	-5.17442600
C	-6.90221000	1.85126000	-2.29845500
Si	3.90755100	8.10062900	-2.77173900
C	3.37446900	9.73818900	-2.00121200
C	4.59266000	8.37506200	-4.50854500
C	5.18563800	7.23735500	-1.68269300
H	-8.87060300	5.32239000	6.08159000
H	-6.90108600	6.85011900	6.20506500
H	-4.75956400	6.24671400	5.06093000
H	-8.72347700	3.17044000	4.81276700
H	-3.64549700	-2.96903100	-0.35207400
H	1.41216000	-1.47564800	-1.75375500
H	-2.26350600	-4.46440300	-1.69282800
H	4.02905000	-4.60992100	-4.50040000
H	4.16828800	-6.79109000	-5.66499600
H	2.21953700	-8.36392000	-5.61758800
H	0.15126100	-7.73533600	-4.40263600
H	6.91045500	-6.82542900	6.22092600
H	8.87894000	-5.29694300	6.09078200
H	8.72946700	-3.14916300	4.81498600
H	4.76756500	-6.22712200	5.07669400
H	3.64150100	2.97341000	-0.36071900
H	2.25612800	4.46460800	-1.70232300
H	-1.41221800	1.46663900	-1.76212300
H	-0.16609800	7.72657500	-4.41697400
H	-2.23498000	8.34815300	-5.63471800
H	-4.17895200	6.76947100	-5.68296100
H	-4.03444500	4.58970600	-4.51641400
H	1.24267900	4.26571600	4.89366400
H	1.54044700	6.01813200	5.04868700
H	-0.06069100	5.34123500	5.45557200
H	-0.89543900	7.38475100	1.73642700

H	0.15045600	8.02223400	3.03458500
H	-1.44439400	7.32207600	3.42847800
H	2.16501600	4.35486300	1.88752000
H	1.39827200	5.50495700	0.75938600
H	2.47448900	6.10520000	2.04966200
H	-10.28712900	-0.53218800	0.56898700
H	-11.31922500	-1.61573000	1.54088200
H	-10.60651800	-0.15545300	2.27919000
H	-8.26327400	-3.28408800	4.11792800
H	-10.01898700	-3.38758500	3.81580300
H	-9.31999600	-1.91649100	4.54546900
H	-8.40953600	-2.93852800	-0.18426800
H	-7.67720600	-3.93116800	1.10009000
H	-9.42764300	-4.04890300	0.77228800
H	-4.13937200	-7.93677900	-0.27250300
H	-4.91264700	-9.42188700	-0.88468800
H	-3.13820500	-9.33888600	-0.72351100
H	-3.58540200	-9.15660900	-5.01568200
H	-4.54343900	-10.21654000	-3.94868300
H	-2.77534900	-10.11198700	-3.74747500
H	-5.58996000	-6.25935700	-2.50505600
H	-5.37774600	-6.68979400	-4.22142800
H	-6.35306700	-7.73202900	-3.15283000
H	7.03298100	-2.91387900	-2.50115100
H	7.85688000	-1.34043600	-2.33202500
H	6.59993400	-1.81611000	-1.16383000
H	4.48822800	1.21977300	-3.34404400
H	6.23389000	1.33894100	-3.00037900
H	5.11293400	0.82968000	-1.71462500
H	6.23216400	-2.16742300	-5.46043200
H	5.32351400	-0.66956500	-5.78361600
H	7.03786500	-0.58570300	-5.29840400
H	10.58788900	0.26073500	2.67193200
H	11.18480400	1.89884900	3.05424500
H	9.98652900	1.11004000	4.11599000
H	7.55689800	4.10941400	2.17713900
H	9.23319200	4.38050800	2.72739300
H	8.05695100	3.57473300	3.80002500
H	9.71955200	1.21789900	-0.19119500
H	8.61803500	2.60657000	-0.37004500
H	10.30111100	2.86672700	0.16355900
H	-1.40458200	-5.47771700	0.77577700
H	-2.47007400	-6.09681500	2.06560400
H	-2.16724700	-4.34333700	1.92224100
H	0.08905400	-5.35845000	5.46208200
H	-1.51516400	-6.03201500	5.06188300
H	-1.21870200	-4.27824100	4.91979000
H	0.89544200	-7.36930000	1.71924400
H	1.45695500	-7.32146600	3.40771700
H	-0.14083300	-8.01824900	3.01933300
H	-5.13417000	-0.82280700	-1.69556500
H	-6.22943400	-1.36019100	-2.99226900
H	-4.47808600	-1.24484500	-3.30410300
H	-6.18419400	2.09367300	-5.52498200
H	-6.99163100	0.51470700	-5.34484400
H	-5.26834200	0.59040100	-5.79853400
H	-6.63593100	1.83186600	-1.22970000
H	-7.04113600	2.90267100	-2.59706000
H	-7.86937500	1.33346000	-2.41382400
H	2.95285500	9.58597000	-0.99476700
H	4.23477400	10.42254800	-1.91070600

H	2.60812100	10.23641600	-2.61656700
H	4.88630400	7.42173200	-4.97647400
H	5.48199200	9.02677700	-4.47622800
H	3.84445500	8.85461000	-5.16002500
H	4.78702600	7.04475100	-0.67342300
H	5.49702600	6.27341200	-2.11702800
H	6.08720600	7.86342400	-1.57463500

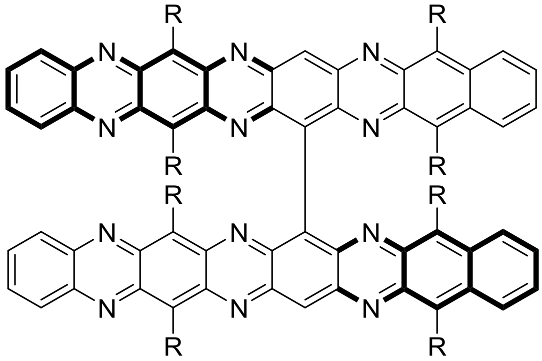

C	-2.80675300	-8.70655600	5.49957300
C	-1.38732700	-8.56981100	5.68941100
C	-0.70407800	-7.51197300	5.15681100
C	-1.40138400	-6.51276200	4.39280900
C	-2.84270700	-6.65131300	4.20068900
C	-3.51423500	-7.78285300	4.78142700
N	-0.73957200	-5.48980500	3.87671500
C	-1.42366200	-4.56855900	3.16420100
C	-2.86515400	-4.70600500	2.97480800
N	-3.52749800	-5.75688600	3.50693400
C	-0.71352100	-3.47059100	2.60296800
C	-1.44154500	-2.49524200	1.84153800
C	-2.89360700	-2.62323100	1.67102400
C	-3.59708100	-3.74055300	2.23490300
N	-0.78297200	-1.47785300	1.29044800
C	-1.46159100	-0.56732100	0.57757500
C	-2.91797200	-0.66256200	0.46076200
N	-3.57964700	-1.70131900	0.99995300
C	-0.76409800	0.50335300	-0.04521200
C	-1.50051100	1.48747100	-0.74864200
C	-2.96103600	1.41553500	-0.79936400
C	-3.63621400	0.33893400	-0.21144000
N	-0.85172700	2.47750800	-1.39114000
C	-1.55632100	3.41062000	-2.01741200
C	-3.02060100	3.37543300	-2.00709000
N	-3.67153500	2.38322200	-1.41558400
C	-0.87256500	4.47225200	-2.71306900
C	-1.62785400	5.48500500	-3.34787400
C	-3.08230400	5.46259200	-3.31288500
C	-3.77061000	4.42180500	-2.65427400
C	-0.98496700	6.56598600	-4.04208800
C	-1.70888200	7.55249300	-4.65437600
C	-3.13826400	7.53013700	-4.61775500
C	-3.79700500	6.52105300	-3.97016700
C	-4.99595800	-3.86886700	2.05638900
C	-6.20648600	-3.97640000	1.89794000
C	0.68461500	-3.36066600	2.79823400
C	1.89172300	-3.32107100	3.00985500
C	0.54808600	4.49402200	-2.75776900
C	1.76703700	4.58344800	-2.85779600
C	-5.19001300	4.37418400	-2.61504800
C	-6.41498200	4.34459000	-2.58606500

C	2.28926300	-8.37307000	-5.69625800
C	3.71547400	-8.35691400	-5.50860100
C	4.32084200	-7.36361600	-4.79001800
C	3.53250900	-6.31151700	-4.20670700
C	2.08433100	-6.32797400	-4.39688400
C	1.49717400	-7.39536100	-5.16128100
N	4.11835900	-5.34992900	-3.51222800
C	3.34791700	-4.37675200	-2.97793900
C	1.89981000	-4.39391900	-3.16590300
N	1.31737300	-5.38237900	-3.87875000
C	3.97336200	-3.33976600	-2.23733900
C	3.15525300	-2.30435700	-1.67160500
C	1.69778800	-2.33118000	-1.84197900
C	1.07723000	-3.37820900	-2.60339800
N	3.73945800	-1.31580300	-0.99904200
C	2.97130700	-0.35324800	-0.45957600
C	1.51319800	-0.41156700	-0.57861200
N	0.93503300	-1.38921600	-1.29119800
C	3.57927700	0.71731500	0.21491200
C	2.79400600	1.71761700	0.80086400
C	1.33406800	1.63891100	0.74320900
C	0.70623400	0.58127000	0.04090800
N	3.39846500	2.75168100	1.42224600
C	2.64631000	3.67252200	2.00907200
C	1.18579100	3.56105000	2.00637800
N	0.58386600	2.55965400	1.37843100
C	3.28243900	4.78721500	2.66390700
C	2.48759600	5.75503000	3.31385300
C	1.03764300	5.63444100	3.33197800
C	0.39283700	4.55160200	2.69091700
C	3.08703800	6.87872600	3.97799600
C	2.32492800	7.81858300	4.61624000
C	0.89993100	7.70067800	4.63587100
C	0.28365000	6.64763900	4.01665500
C	-0.32477900	-3.41688600	-2.79790000
C	-1.52946100	-3.50419600	-3.00891600
C	5.37802700	-3.31750600	-2.05975400
C	6.59286200	-3.29126800	-1.90003900
C	4.70011500	4.87920700	2.64225000
C	5.92233300	4.97096800	2.63148700
C	-1.02378600	4.43576000	2.71732800
C	-2.24705800	4.41046000	2.80051100
Si	-8.03257300	-4.14524800	1.66710200
C	-8.37160900	-5.76091300	0.74853200
C	-8.65408100	-2.67215800	0.66335800
C	-8.85036000	-4.17632000	3.36968400
Si	3.68510100	-3.42863000	3.46421600
C	4.36034000	-5.05104700	2.77163100
C	3.78611000	-3.42765300	5.35170400
C	4.63828000	-1.96612300	2.75514100
Si	3.59037100	4.80901300	-3.11554200
C	4.55191000	3.44295000	-2.24711300
C	4.07222400	6.49732400	-2.41744000
C	3.90910600	4.75626100	-4.97803400
Si	-8.26010800	4.22840300	-2.50384100
C	-8.78468300	2.56727900	-3.23062200
C	-9.00068200	5.65049800	-3.50238600
C	-8.78293600	4.35310900	-0.69351400
Si	-3.30215800	-3.79570200	-3.46332100
C	-4.39769100	-2.43109500	-2.76485400
C	-3.81240500	-5.47289400	-2.75991400

C	-3.40021800	-3.81809600	-5.35080800
Si	8.42387200	-3.23691000	-1.65328900
C	9.20803300	-4.65196200	-2.62694800
C	8.78292600	-3.42801600	0.19160300
C	9.06212400	-1.57234800	-2.27698100
Si	7.77115000	5.04367800	2.58969500
C	8.38303200	3.83749500	1.27402900
C	8.42780800	4.55643900	4.29232900
C	8.29559000	6.80866300	2.16703600
Si	-4.08717800	4.47277000	3.03035500
C	-4.90153700	2.97121900	2.23846600
C	-4.72164900	6.06167000	2.22905200
C	-4.42249200	4.49918200	4.89061400
H	-3.31846800	-9.56584400	5.94042300
H	-0.85608900	-9.32850200	6.26964600
H	0.37246000	-7.38521800	5.28578900
H	-4.59130300	-7.86329900	4.62296700
H	-4.72223500	0.27820000	-0.27415800
H	0.10479400	6.58069900	-4.06706100
H	-1.19497600	8.36425500	-5.17506400
H	-3.70370700	8.32467900	-5.11092800
H	-4.88705700	6.49753100	-3.93925800
H	1.84164500	-9.18368400	-6.27681900
H	4.31578300	-9.15571500	-5.95146000
H	5.40055900	-7.32810200	-4.63308400
H	0.41306300	-7.38474600	-5.28865900
H	4.66548100	0.77032800	0.28105200
H	4.17439400	6.96211800	3.95973800
H	2.80380400	8.66496200	5.11479900
H	0.30269500	8.45829100	5.14919400
H	-0.80249100	6.55531800	4.02894800
H	-7.99216200	-6.62544400	1.31658800
H	-9.45473700	-5.90185300	0.59395200
H	-7.88300000	-5.76562100	-0.23906500
H	-8.44031600	-1.72354700	1.18162500
H	-9.74368800	-2.73713100	0.50429500
H	-8.16620700	-2.63283000	-0.32384100
H	-9.94414400	-4.28397200	3.27490300
H	-8.47513500	-5.01852100	3.97299100
H	-8.64633300	-3.24648500	3.92459300
H	4.30238300	-5.06614100	1.67149000
H	5.41788700	-5.18258600	3.05683100
H	3.79140900	-5.91330400	3.15459400
H	3.38124200	-2.49195300	5.76979300
H	4.83339200	-3.52437800	5.68475200
H	3.21116000	-4.26491800	5.77866600
H	4.62753500	-1.97324700	1.65372300
H	4.21580000	-1.00687200	3.09448200
H	5.69095600	-2.00784300	3.08319200
H	4.23376300	2.44541300	-2.58928700
H	5.62908900	3.54652200	-2.46244700
H	4.42039000	3.48642900	-1.15455600
H	3.51024600	7.30982100	-2.90563600
H	5.14778000	6.68632200	-2.57385200
H	3.87337600	6.54893900	-1.33496200
H	3.60811900	3.78559800	-5.40396800
H	3.34710900	5.54487100	-5.50393500
H	4.98151000	4.90298600	-5.19114900
H	-8.33395100	1.73248700	-2.67040400
H	-9.88118400	2.45330400	-3.19176400
H	-8.46947500	2.47674900	-4.28254200

H	-8.68647300	6.62673100	-3.09882100
H	-10.10274300	5.61237300	-3.47619200
H	-8.68563100	5.60228500	-4.55736200
H	-8.33152000	3.54390300	-0.09727700
H	-8.46751500	5.31329700	-0.25443800
H	-9.87929800	4.27789700	-0.59788000
H	-4.07046700	-1.43676700	-3.10837200
H	-5.44010200	-2.57729300	-3.09568200
H	-4.38969700	-2.43136200	-1.66336500
H	-3.15854400	-6.27635700	-3.13506800
H	-4.85034400	-5.71264000	-3.04684200
H	-3.75678800	-5.47436100	-1.65953400
H	-3.09119800	-2.84924500	-5.77515900
H	-2.74303300	-4.59602800	-5.77130300
H	-4.43187700	-4.02232900	-5.68402000
H	8.98678100	-4.56141800	-3.70262800
H	10.30407000	-4.65373900	-2.50266200
H	8.82408700	-5.62657300	-2.28496200
H	8.30564700	-2.62171600	0.77144200
H	9.86859300	-3.39161000	0.38376100
H	8.40151600	-4.38934500	0.57180900
H	8.84401700	-1.44057800	-3.34899900
H	8.58982700	-0.73949600	-1.73172700
H	10.15399100	-1.49660900	-2.13910100
H	7.98955800	4.10314900	0.27975700
H	9.48466500	3.84321400	1.21897900
H	8.05676600	2.80984200	1.50066000
H	8.06066300	5.24264100	5.07247500
H	9.53049200	4.58417100	4.30634000
H	8.10931700	3.53678600	4.56234300
H	9.39508700	6.88865200	2.12815300
H	7.89765300	7.11794100	1.18713900
H	7.93071600	7.52469800	2.92117000
H	-4.48894600	2.03055100	2.63633200
H	-5.98536300	2.98021400	2.44497900
H	-4.76630900	2.96895400	1.14553900
H	-4.24644700	6.95281700	2.66984900
H	-5.81218900	6.15334900	2.36814000
H	-4.51742200	6.06699800	1.14639200
H	-4.03430800	3.58942000	5.37627300
H	-3.94632400	5.36860900	5.37200200
H	-5.50665000	4.55320000	5.08728400

2.4 Comparison of 4 with 4-H_4 and 3-H_2

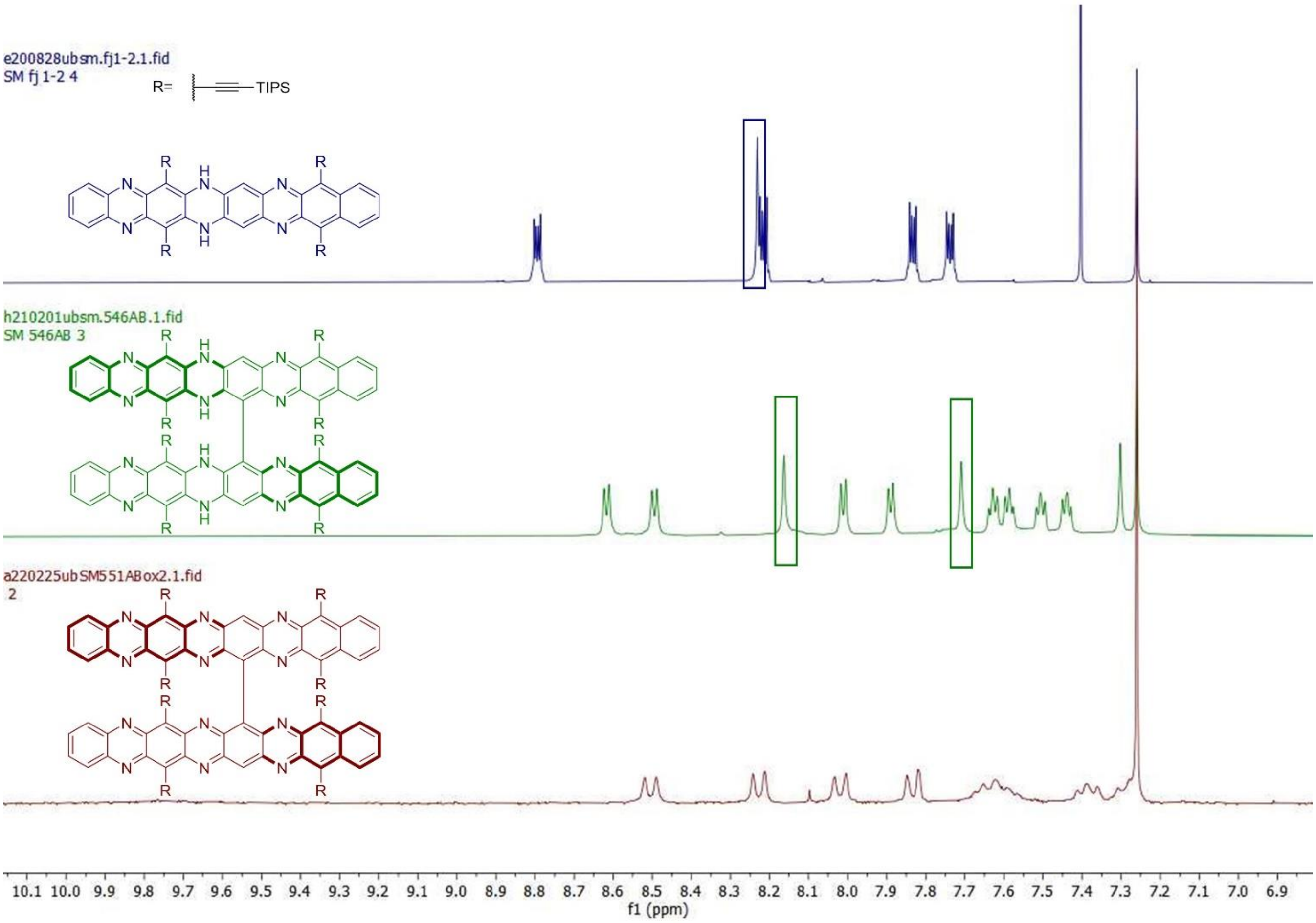

Figure S10. Comparison of the aromatic region of the ^{1}H NMR spectra of **3-H_2** (Top), **4-H_4** (middle) and **4** (bottom). Resonances originating from amines are highlighted in boxes and are absent for **4**.

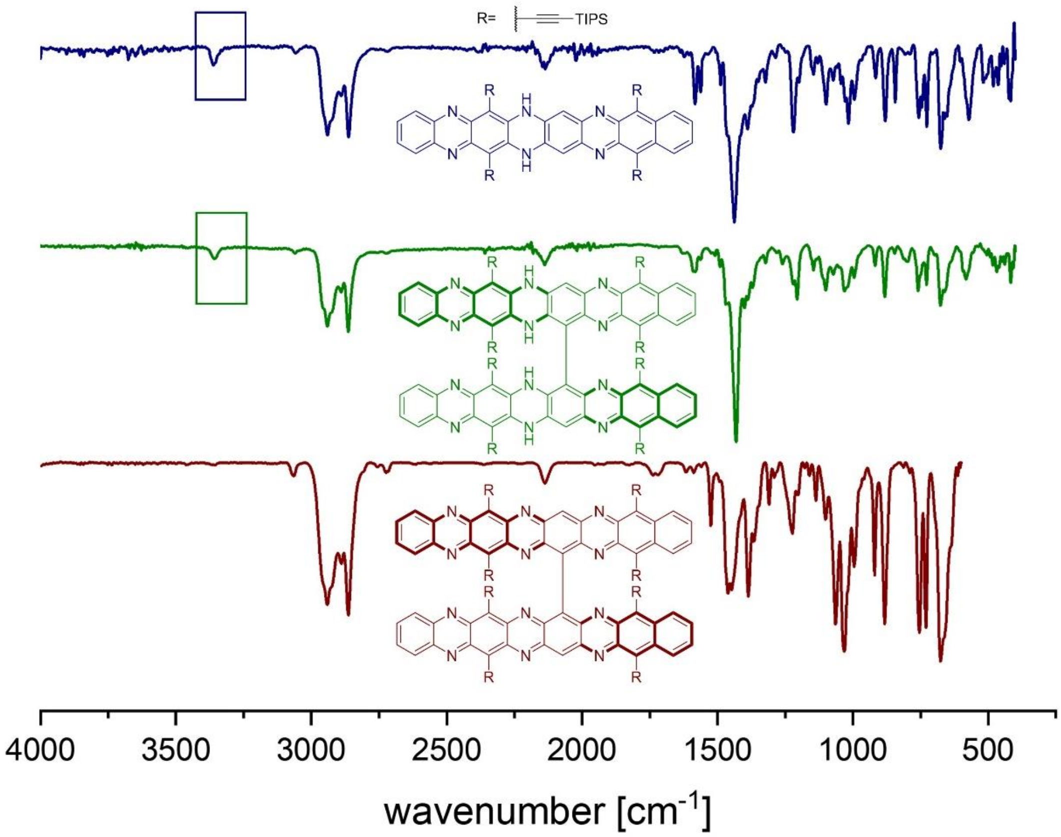

Figure S11. Comparison of the IR spectra of **3-H₂** (top), **4-H₄** (middle) and **4** (bottom). The characteristic bands of the NH-vibration are highlighted in boxes and are absent for **4**.

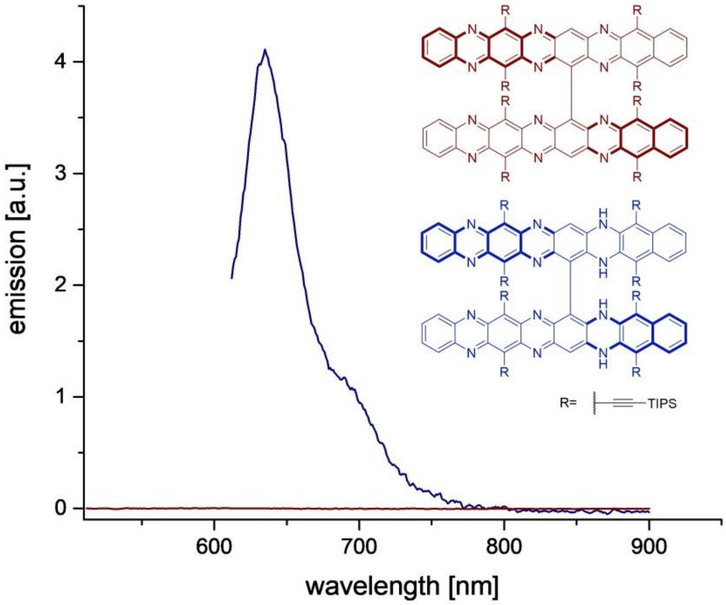

Figure S12. Emission spectra of **4-H₄** (blue; excitation at 590 nm) and **4** (brown; excitation at 491 nm) in DCM (c = 3 x 10^{-6} mol/L).

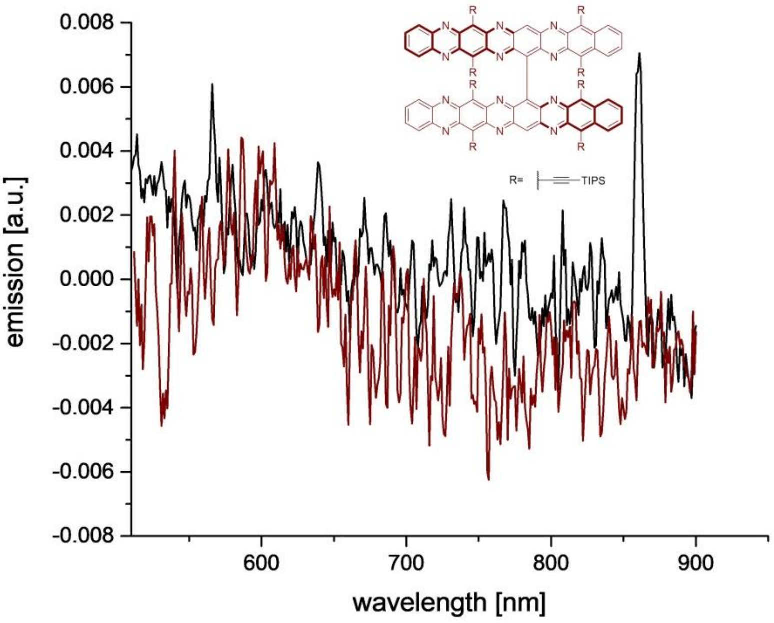

Figure S13. Emission spectra of **4** (brown; excitation at 491 nm) in DCM and a pure DCM sample (black; excitation at 491 nm).

2.5 NMR Spectroscopy

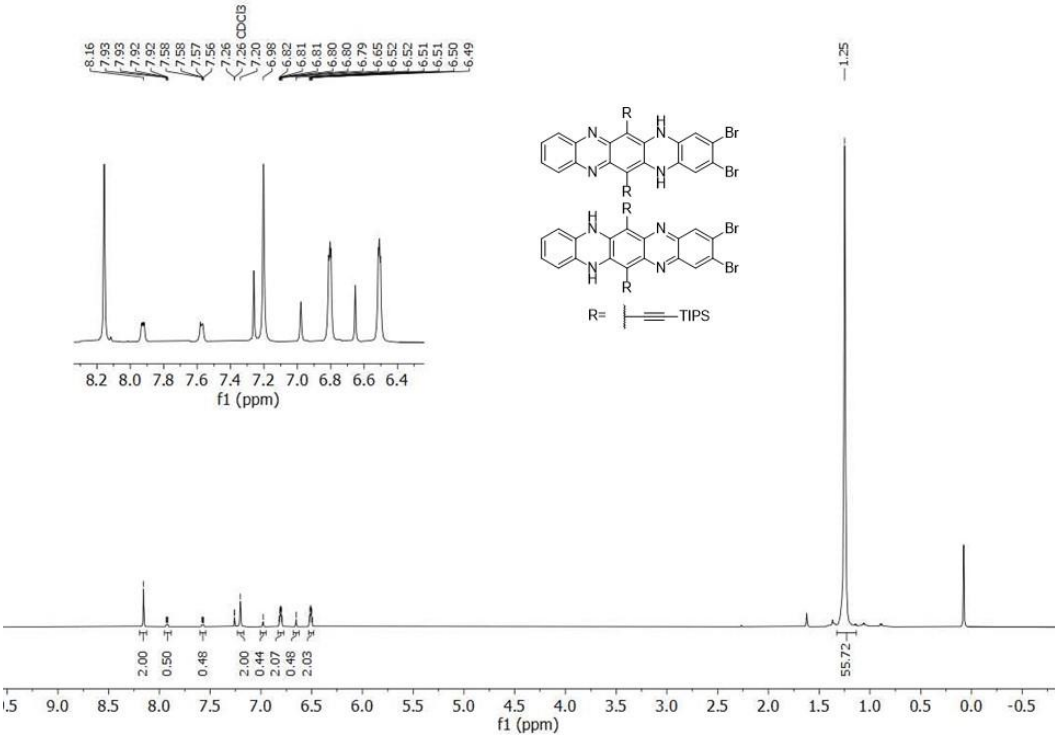

Figure S14. ^{1}H-NMR spectrum (600 MHz, $CDCl_3$, 295 K) of **1.** Both tautomers were observed.

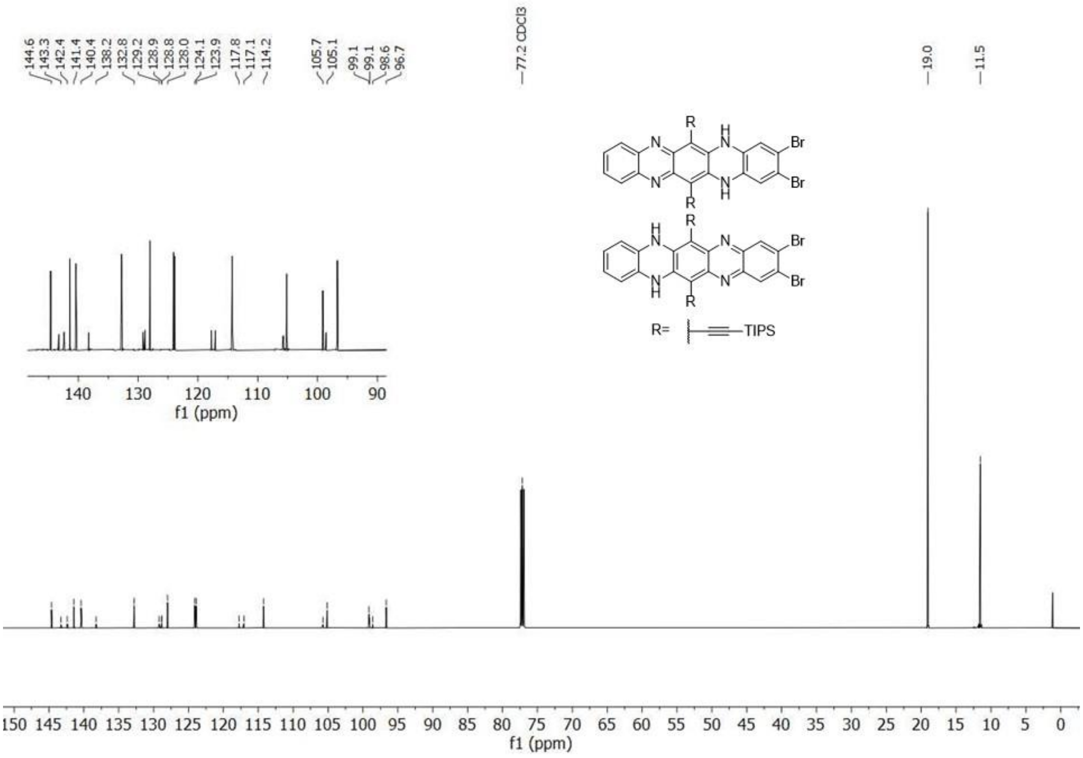

Figure S15. $^{13}C\{^{1}H\}$ NMR spectrum (151 MHz, $CDCl_3$, 295 K) of **1.** Both tautomers were observed.

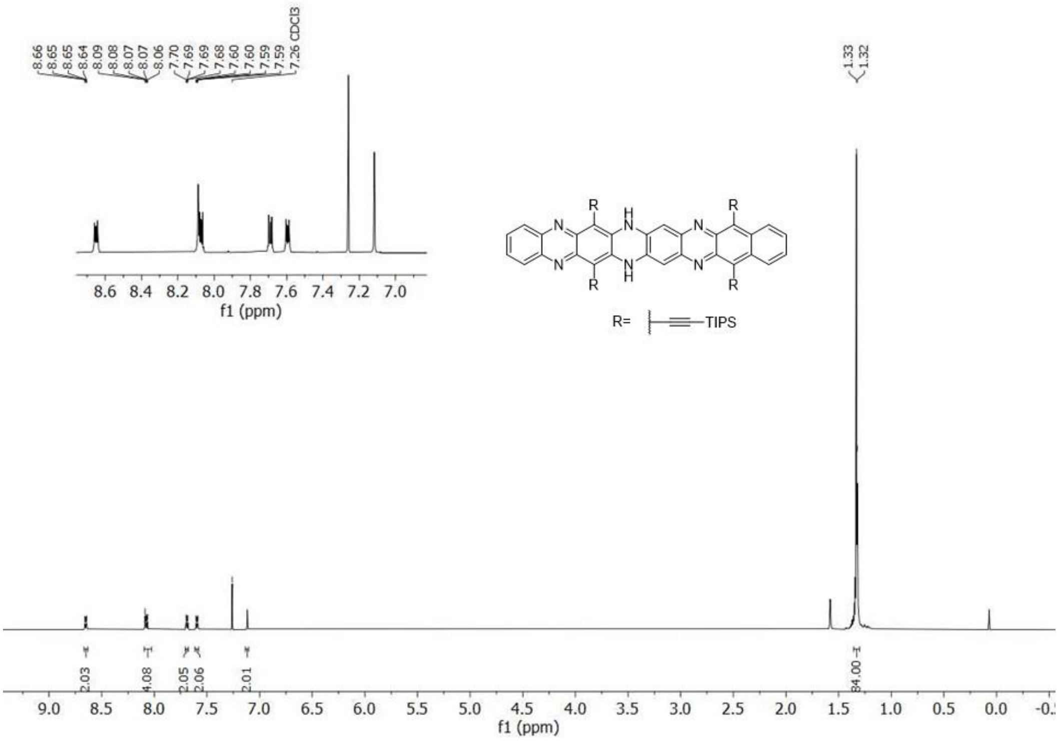

Figure S16. ^{1}H NMR spectrum (600 MHz, $CDCl_3$, 295 K) of **3-H$_2$**.

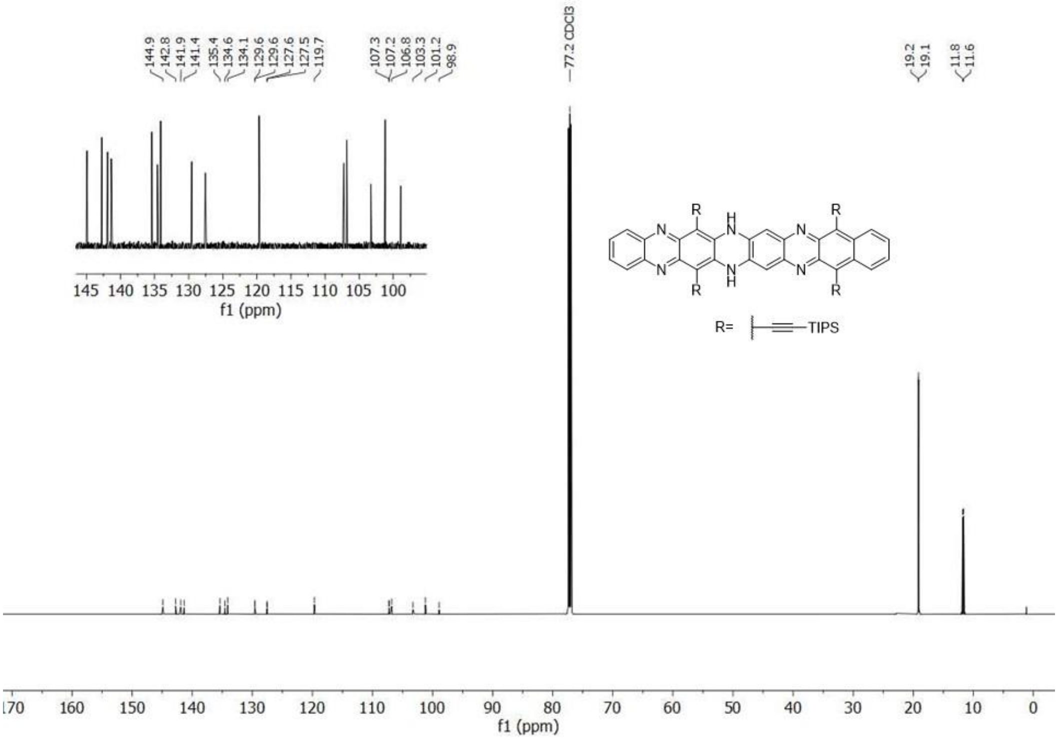

Figure S17. $^{13}C\{^{1}H\}$ NMR spectrum (151 MHz, $CDCl_3$, 295 K) of **3-H$_2$**.

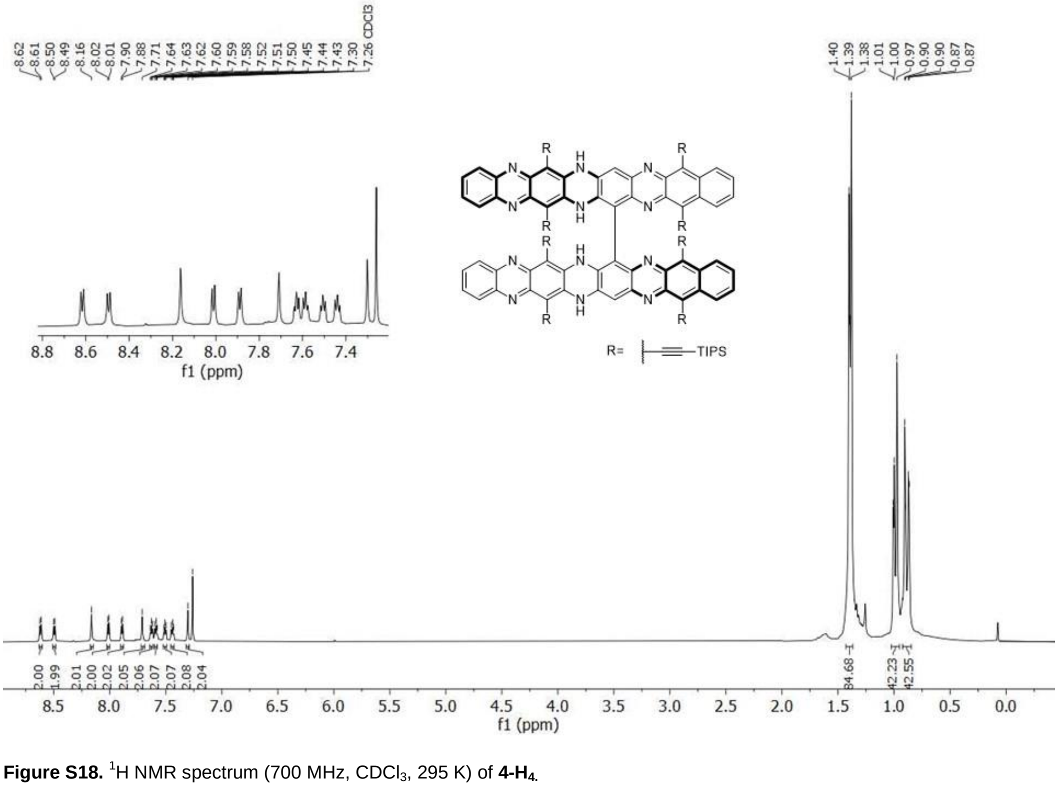

Figure S18. ^{1}H NMR spectrum (700 MHz, $CDCl_3$, 295 K) of **4-H$_4$**.

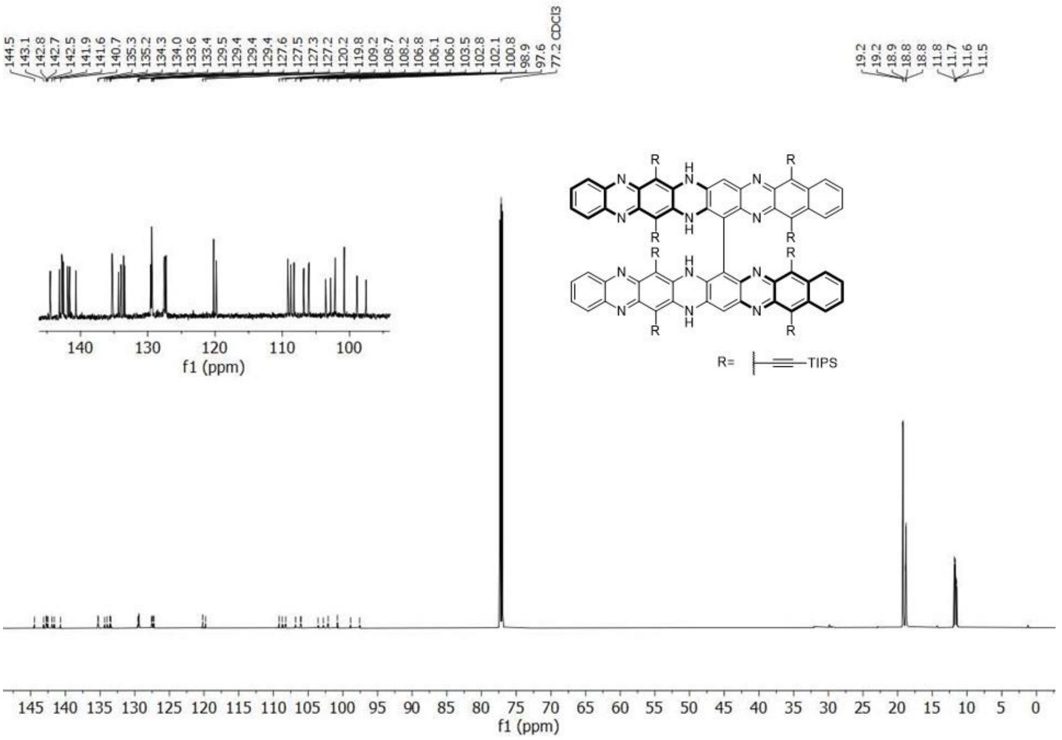

Figure S19. $^{13}C\{^1H\}$ NMR spectrum (176 MHz, $CDCl_3$, 295 K) of **4-H$_4$**.

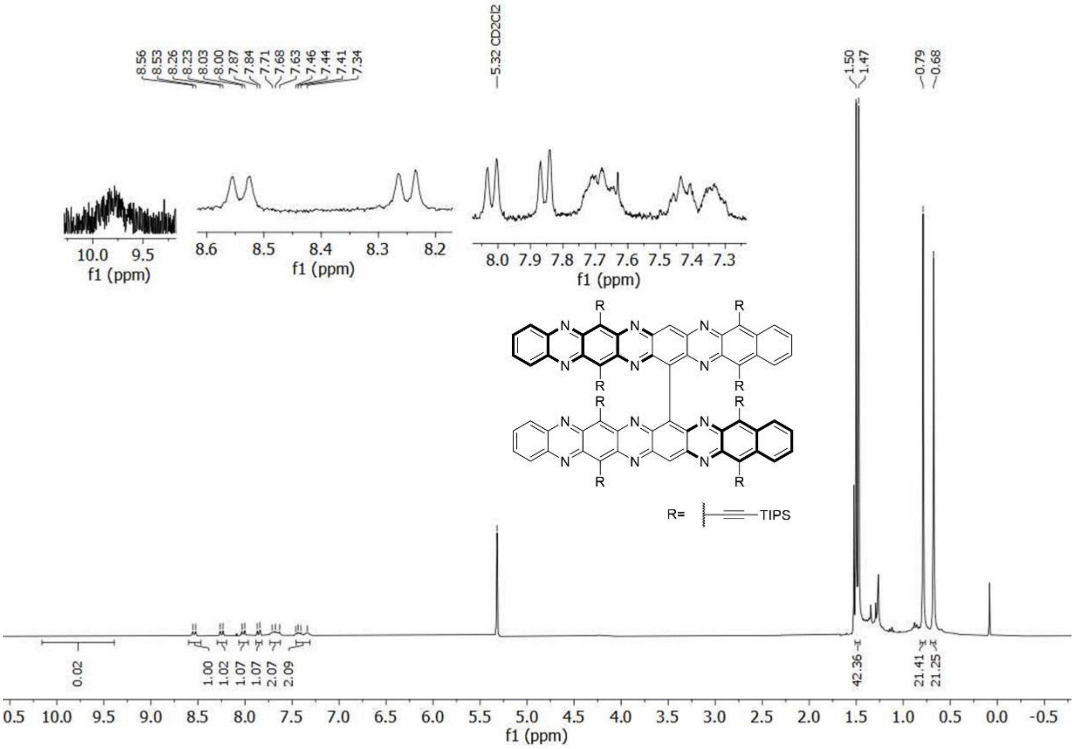

Figure S20. ^1H NMR spectrum (300 MHz, CD_2Cl_2, 295 K) of **4**. Note that the resonance belonging to the central proton on the octacene backbone between 9.5 and 10.0 ppm is broadened, most likely due to the diradical character of **4** (see EPR spectrum).

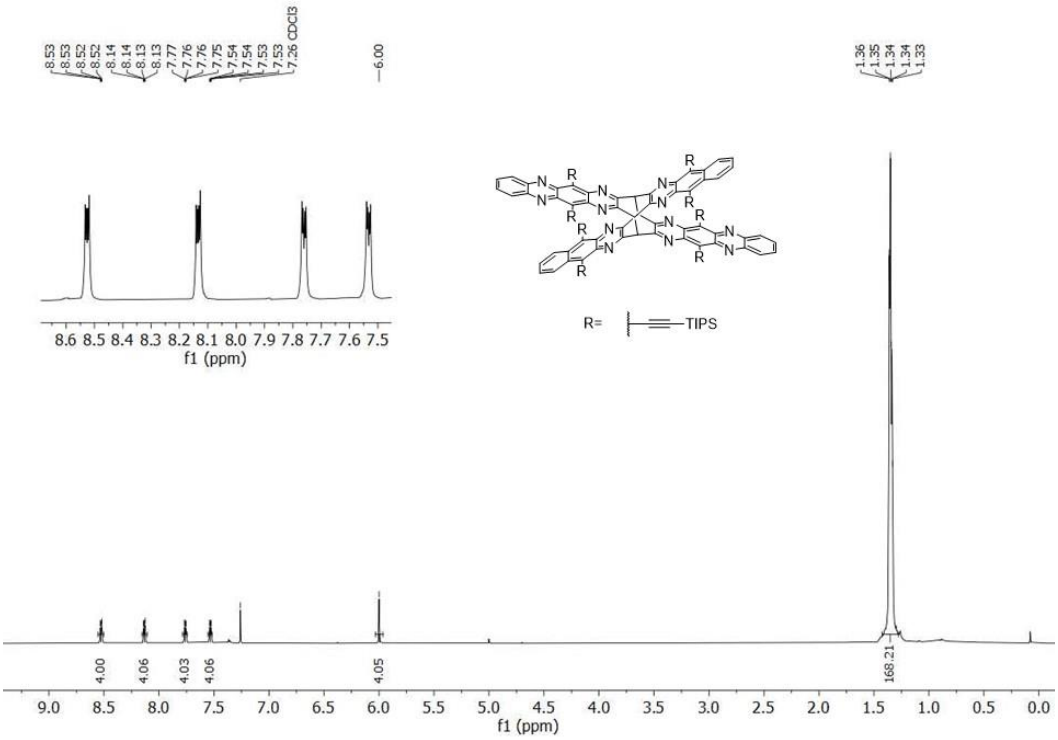

Figure S21. ^{1}H NMR spectrum (700 MHz, $CDCl_3$, 295 K) of **5.**

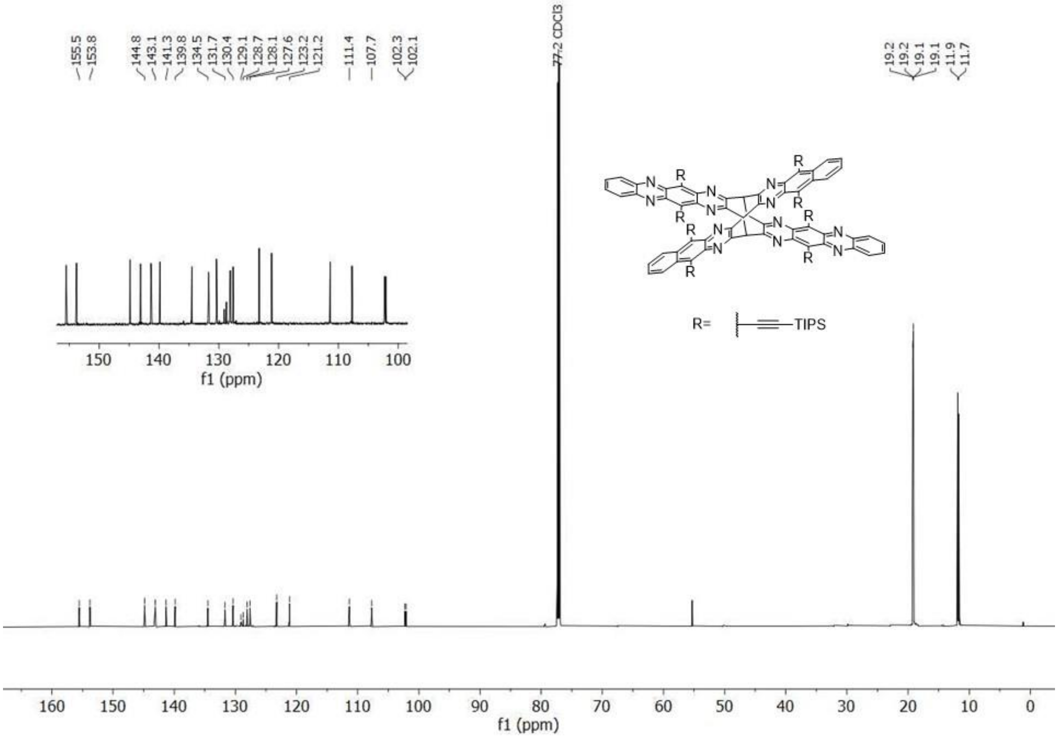

Figure S22. $^{13}C\{^{1}H\}$ NMR spectrum (176 MHz, $CDCl_3$, 295 K) of **5.**

2.6 UV/vis Stability Studies

All UV/vis stability studies were performed using dilute solutions (10^{-5} mol L^{-1}, 3.00 mL anhydrous dichloromethane) of the respective acene in quartz cuvettes at room temperature. Stability studies were carried out under ambient conditions.

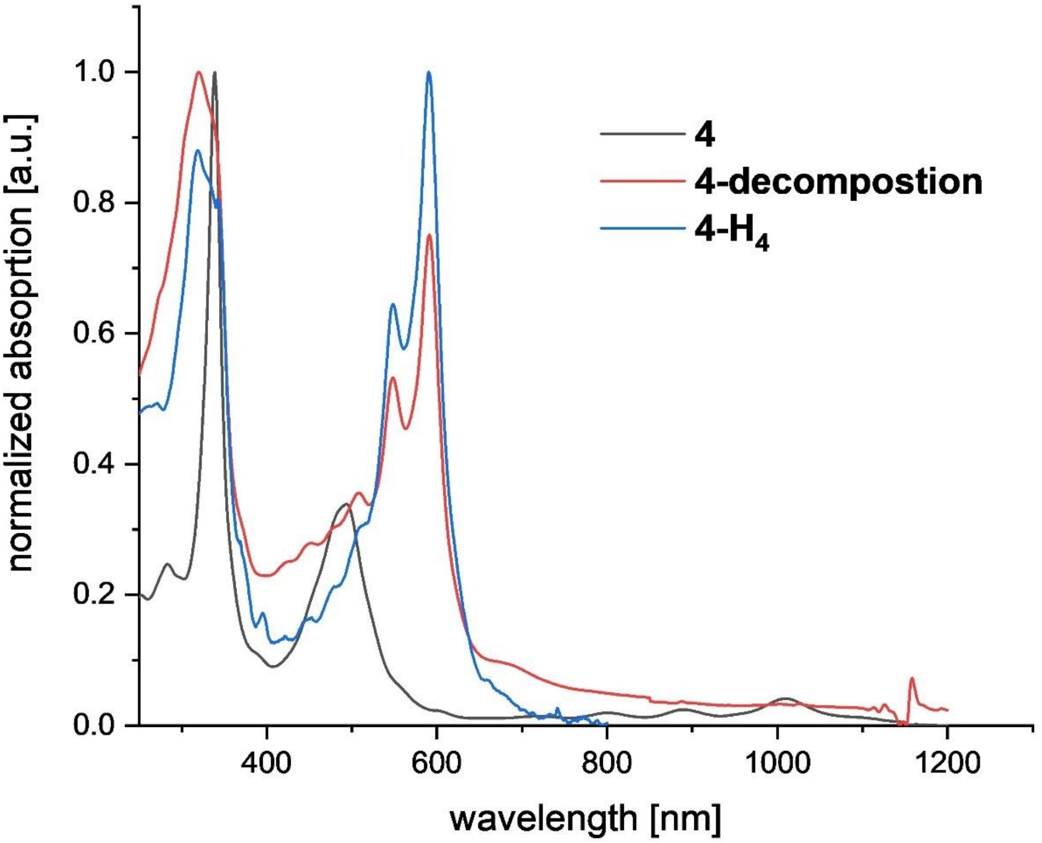

Figure S23. Absorption spectra of 4 (black), 4 after 35 d in DCM solution (red) and 4-H4 in DCM.

2.7 Cyclic Voltammetry

The cyclic voltammetry (CV) experiments were carried out using a platinum working electrode, a platinum wire auxiliary electrode, a silver wire reference electrode, a 0.1 mol L^{-1} NBu_4PF_6 solution in degassed, dry DCM, and ferrocene/ferrocenium as the reference redox system and internal standard (−5.1 eV)[S9] at room temperature and 0.2 V s^{-1} or 0.5 V s^{-1}. To determine the first reduction potentials ($E_{(0/-)}$) of the samples and the first oxidation potential of ferrocene, the half-wave potentials were used.

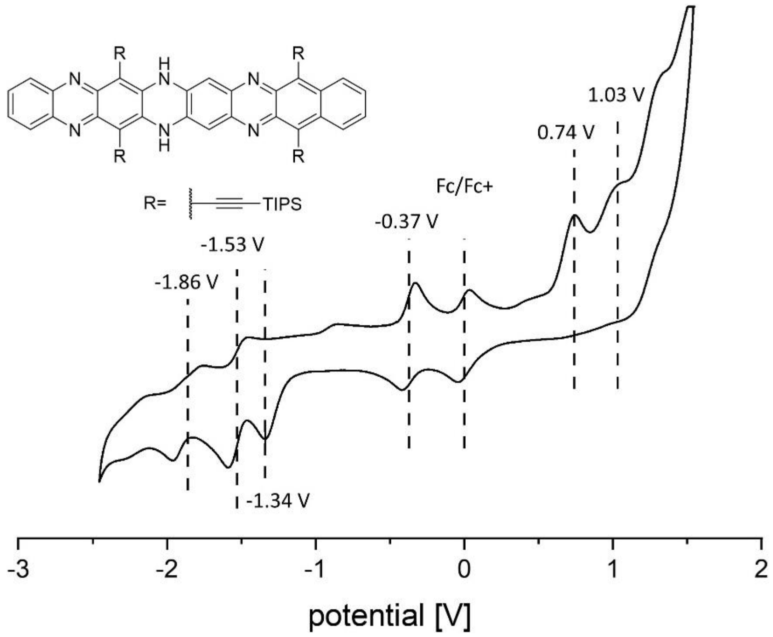

Figure S24. CV spectrum of **3-H2** containing ferrocene as internal standard.

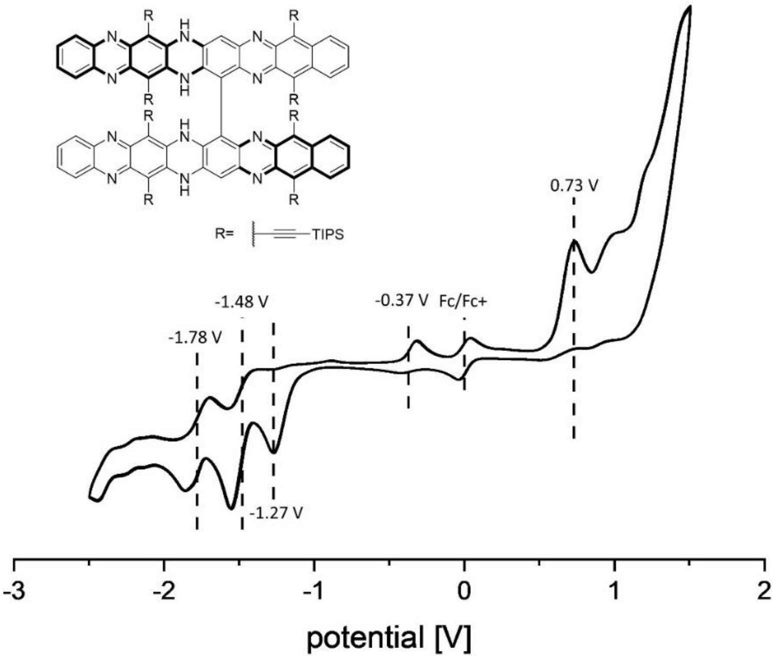

Figure S25. CV spectrum of **4-H4**. Here the CV spectrum without ferrocen is shown for clarity.

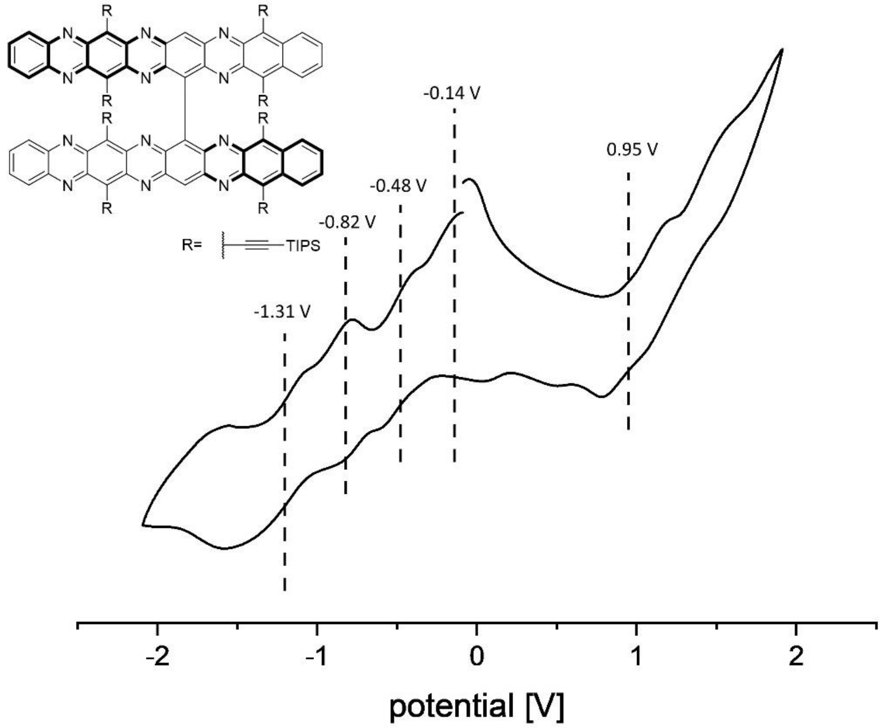

Figure S26. CV spectrum of **4**. The spectrum without ferrocene is shown due to decomposition of the compound in the ferrocene sample. We noticed also a deposition on the electrodes in the measurement without ferrocene. That is why the quality of the spectrum is not that high.

2.8 EPR Spectroscopy

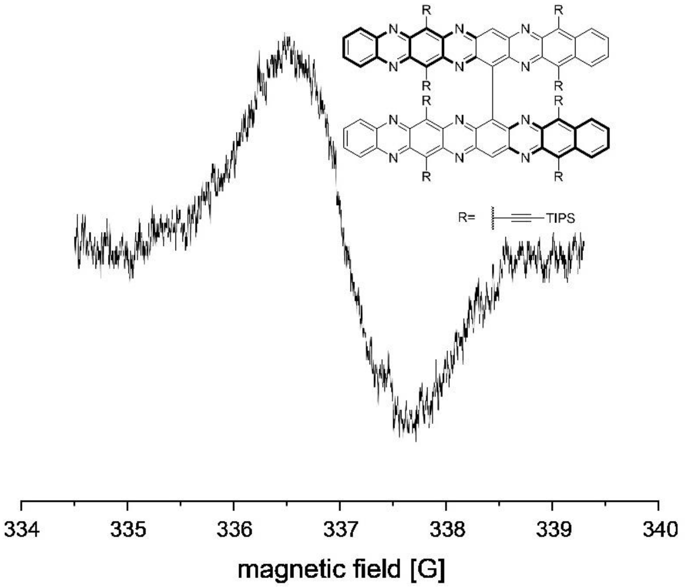

Figure S27. EPR spectrum of **4** in DCM (9.45 GHz, r.t.).

2.9 Crystallogrpahic Data

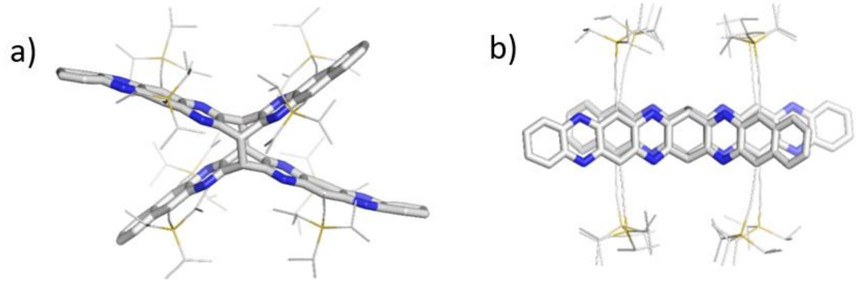

Figure S28. X-ray structure of the butterfly dimer **5**. a) Front view; b) top view.

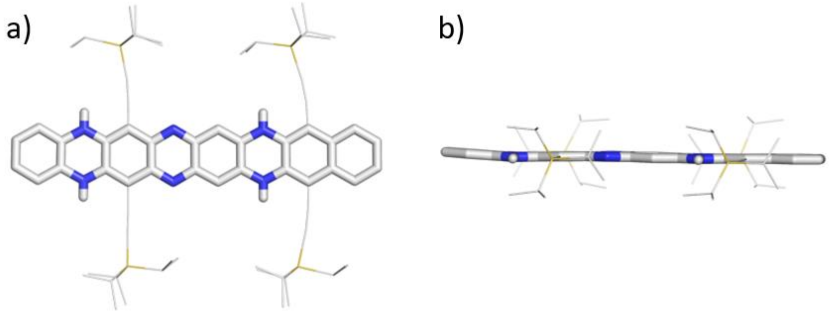

Figure S29. X-ray structure of **3-H$_4$**. a) front view; b) top view. A pure bulk sample for further characterization of **3-H$_4$** was not isoable due to oxidation to **3H$_2$** under ambient conditions. The crystal was grown out of a mixture of **3-H$_4$** and **3-H$_2$**.

Table S1. Crystal structure, crystal data and structure refinement of **3-H_4** (CCDC: 2116992).

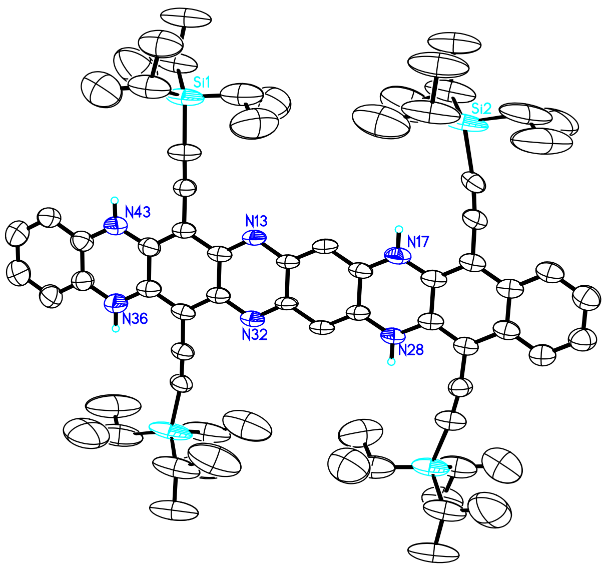

Identification code	mai37	
Empirical formula	$C_{72}H_{98}N_6Si_4$	
Formula weight	1159.92	
Temperature	200(2) K	
Wavelength	0.71073 Å	
Crystal system	triclinic	
Space group	$P\bar{1}$	
Z	1	
Unit cell dimensions	a = 10.3543(5) Å	α = 82.7618(11) deg.
	b = 10.5730(5) Å	β = 87.6908(12) deg.
	c = 17.7937(8) Å	γ = 64.4354(10) deg.
Volume	1743.06(14) $Å^3$	
Density (calculated)	1.11 g/cm^3	
Absorption coefficient	0.13 mm^{-1}	
Crystal shape	brick	
Crystal size	0.126 x 0.083 x 0.043 mm^3	
Crystal colour	red	
Theta range for data collection	1.2 to 28.0 deg.	
Index ranges	$-13\leq h\leq 13$, $-13\leq k\leq 13$, $-22\leq l\leq 22$	
Reflections collected	32117	
Independent reflections	7932 (R(int) = 0.0282)	
Observed reflections	4069 ($I > 2\sigma(I)$)	
Absorption correction	Semi-empirical from equivalents	
Max. and min. transmission	0.96 and 0.92	
Refinement method	Full-matrix least-squares on F^2	
Data/restraints/parameters	7932 / 1574 / 609	
Goodness-of-fit on F^2	1.03	
Final R indices (I>2sigma(I))	R1 = 0.087, wR2 = 0.239	
Largest diff. peak and hole	0.31 and -0.64 $eÅ^{-3}$	

Table S2. Crystal structure, crystal data and structure refinement of **4-H$_4$** (CCDC: 2116995).

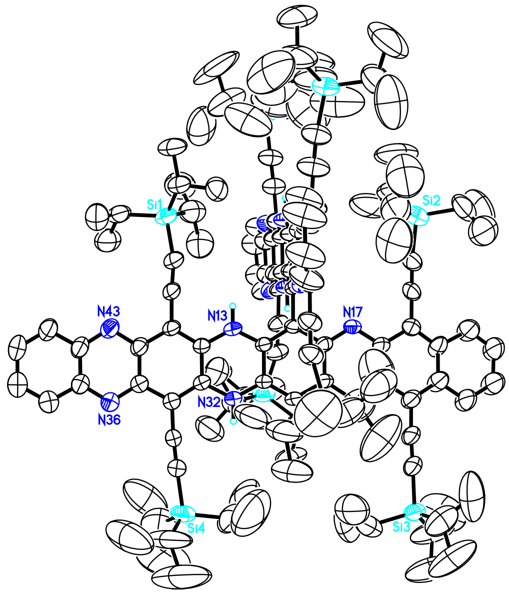

Identification code	mai28	
Empirical formula	$C_{72}H_{96}N_6O_{0.12}Si_4$	
Formula weight	1159.90	
Temperature	200(2) K	
Wavelength	0.71073 Å	
Crystal system	monoclinic	
Space group	C2/c	
Z	8	
Unit cell dimensions	a = 20.2227(6) Å	α = 90 deg.
	b = 26.9980(8) Å	β = 95.671(2) deg.
	c = 25.8156(8) Å	γ = 90 deg.
Volume	14025.6(7) $Å^3$	
Density (calculated)	1.10 g/cm^3	
Absorption coefficient	0.13 mm^{-1}	
Crystal shape	plank	
Crystal size	0.486 x 0.048 x 0.046 mm^3	
Crystal colour	violet	
Theta range for data collection	1.5 to 21.3 deg.	
Index ranges	$-20 \le h \le 20$, $-27 \le k \le 27$, $-26 \le l \le 26$	
Reflections collected	46614	
Independent reflections	7829 (R(int) = 0.0682)	
Observed reflections	4810 ($I > 2\sigma(I)$)	
Absorption correction	Semi-empirical from equivalents	
Max. and min. transmission	0.96 and 0.84	
Refinement method	Full-matrix least-squares on F^2	
Data/restraints/parameters	7829 / 1598 / 799	
Goodness-of-fit on F^2	1.03	
Final R indices (I>2sigma(I))	R1 = 0.104, wR2 = 0.287	
Largest diff. peak and hole	0.58 and -0.42 $eÅ^{-3}$	

Table S3. Crystal structure, crystal data and structure refinement of **4** (CCDC: 2116993).

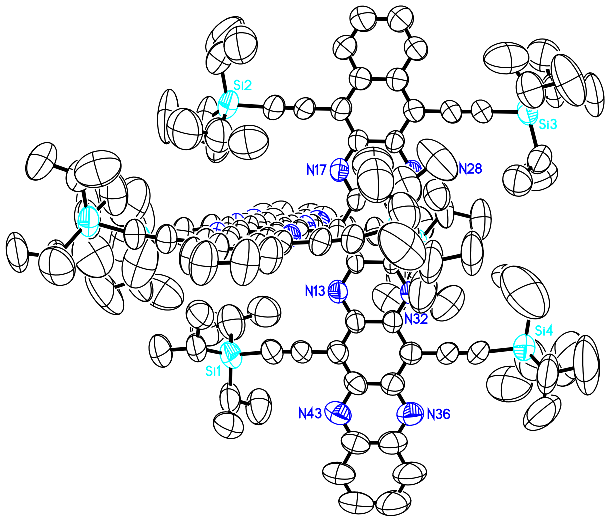

Identification code	mai30	
Empirical formula	$C_{144}H_{186}N_{12}Si_8$	
Formula weight	2309.76	
Temperature	200(2) K	
Wavelength	1.54178 Å	
Crystal system	monoclinic	
Space group	C2/c	
Z	4	
Unit cell dimensions	a = 20.1554(8) Å	α = 90 deg.
	b = 27.2212(10) Å	β = 97.498(3) deg.
	c = 25.8161(11) Å	γ = 90 deg.
Volume	14043.0(10) $Å^3$	
Density (calculated)	1.09 g/cm^3	
Absorption coefficient	1.11 mm^{-1}	
Crystal shape	plank	
Crystal size	0.160 x 0.100 x 0.018 mm^3	
Crystal colour	green	
Theta range for data collection	3.1 to 49.8 deg.	
Index ranges	$-19 \le h \le 19$, $-26 \le k \le 26$, $-25 \le l \le 15$	
Reflections collected	29274	
Independent reflections	7065 (R(int) = 0.0816)	
Observed reflections	4253 ($I > 2\sigma(I)$)	
Absorption correction	Semi-empirical from equivalents	
Max. and min. transmission	1.39 and 0.63	
Refinement method	Full-matrix least-squares on F^2	
Data/restraints/parameters	7065 / 1471 / 739	
Goodness-of-fit on F^2	1.07	
Final R indices (I>2sigma(I))	R1 = 0.116, wR2 = 0.288	
Largest diff. peak and hole	0.35 and -0.25 $eÅ^{-3}$	

Table S4. Crystal structure, crystal data and structure refinement of **5** (CCDC: 2116994).

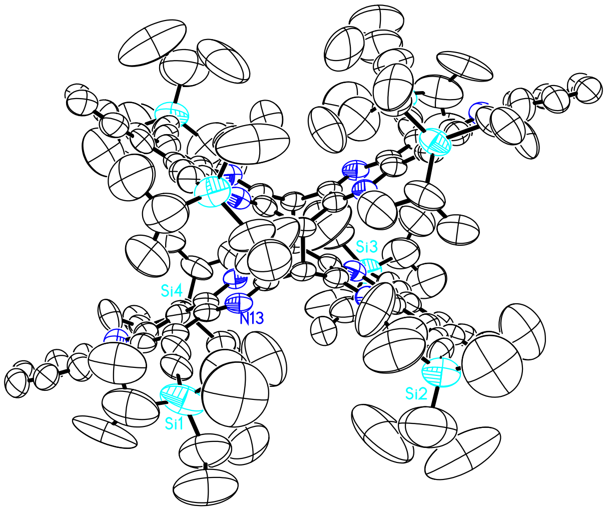

Identification code	mai29	
Empirical formula	$C_{72}H_{94}N_6Si_4$	
Formula weight	1155.89	
Temperature	200(2) K	
Wavelength	1.54178 Å	
Crystal system	monoclinic	
Space group	$P2_1/c$	
Z	4	
Unit cell dimensions	a = 18.1432(10) Å	α = 90 deg.
	b = 30.2157(16) Å	β = 107.756(4) deg.
	c = 13.5085(7) Å	γ = 90 deg.
Volume	7052.7(7) $Å^3$	
Density (calculated)	1.09 g/cm^3	
Absorption coefficient	1.10 mm^{-1}	
Crystal shape	plate	
Crystal size	0.105 x 0.093 x 0.019 mm^3	
Crystal colour	orange	
Theta range for data collection	2.6 to 50.4 deg.	
Index ranges	$-18 \le h \le 17$, $-30 \le k \le 24$, $-11 \le l \le 13$	
Reflections collected	30678	
Independent reflections	7351 (R(int) = 0.1201)	
Observed reflections	4686 ($I > 2\sigma(I)$)	
Absorption correction	Semi-empirical from equivalents	
Max. and min. transmission	1.45 and 0.69	
Refinement method	Full-matrix least-squares on F^2	
Data/restraints/parameters	7351 / 1990 / 776	
Goodness-of-fit on F^2	2.48	
Final R indices (I>2sigma(I))	R1 = 0.178, wR2 = 0.406	
Largest diff. peak and hole	1.02 and -0.54 $eÅ^{-3}$	

2.9 References

[S1] G. R. Fulmer, A. J. Miller, N. H. Sherden, H. E. Gottlieb, A. Nudelman, B. M. Stoltz, J. E. Bercaw, K. I. Goldberg, *Organometallics* **2010,** *29*, 2176-2179.

[S2] Gaussian 16, Revision B.01, M. J. Frisch, G. W. Trucks, H. B. Schlegel, G. E. Scuseria, M. A. Robb, J. R. Cheeseman, G. Scalmani, V. Barone, G. A. Petersson, H. Nakatsuji, X. Li, M. Caricato, A. V. Marenich, J. Bloino, B. G. Janesko, R. Gomperts, B. Mennucci, H. P. Hratchian, J. V. Ortiz, A. F. Izmaylov, J. L. Sonnenberg, D. Williams-Young, F. Ding, F. Lipparini, F. Egidi, J. Goings, B. Peng, A. Petrone, T. Henderson, D. Ranasinghe, V. G. Zakrzewski, J. Gao, N. Rega, G. Zheng, W. Liang, M. Hada, M. Ehara, K. Toyota, R. Fukuda, J. Hasegawa, M. Ishida, T. Nakajima, Y. Honda, O. Kitao, H. Nakai, T. Vreven, K. Throssell, J. A. Montgomery, Jr., J. E. Peralta, F. Ogliaro, M. J. Bearpark, J. J. Heyd, E. N. Brothers, K. N. Kudin, V. N. Staroverov, T. A. Keith, R. Kobayashi, J. Normand, K. Raghavachari, A. P. Rendell, J. C. Burant, S. S. Iyengar, J. Tomasi, M. Cossi, J. M. Millam, M. Klene, C. Adamo, R. Cammi, J. W. Ochterski, R. L. Martin, K. Morokuma, O. Farkas, J. B. Foresman, and D. J. Fox, Gaussian, Inc., Wallingford CT, **2016**.

[S3] B. D. Lindner, J. U. Engelhart, O. Tverskoy, A. L. Appleton, F. Rominger, A. Peters, H. J. Himmel, U. H. F. Bunz. *Angew. Chem. Int. Ed*. **2011**, *50*, 8588-8591.

[S4] M. Fleming, H. McManus, A. Rudolph, W. Chan, J. Ruiz, C. Dockendorff, M. Lautens, *Chem. Eur. J.* **2008**, *14*, 2112-2124.

[S5] S. Miao, S. M. Brombosz, P. v. R. Schleyer, J. I. Wu‡, S. Barlow†, S. R. Marder, K. I. Hardcastle, U. H. F. Bunz *J. Am. Chem. Soc.* **2008**, *130*, 7339–7344.

[S6] P. von Ragué Schleyer, C. Maerker, A. Dransfeld, H. Jiao, N. J. R. van Eikema Hommes, J. Am. Chem. Soc. 1996, 118, 6317–6318.

[S7] a) D. Geuenich, R. Herges, *J. Phys. Chem. A* **2001**, *105*, 3214-3220; b) D. Geuenich, K. Hess, F. Koehler, R. Herges, *Chem. Rev.* **2005**, *105*, 3758-3772.

[S8] Y. Shao, Z. Gan, E. Epifanovsky, A. T. B. Gilbert, M. Wormit, J. Kussmann, A. W. Lange, A. Behn, J. Deng, X. Feng, D. Ghosh, M. Goldey P. R. Horn, L. D. Jacobson, I. Kaliman, R. Z. Khaliullin, T. Kús, A. Landau, J. Liu, E. I. Proynov, Y. M. Rhee, R. M. Richard, M. A. Rohrdanz, R. P. Steele, E. J. Sundstrom, H. L. Woodcock III, P. M. Zimmerman, D. Zuev, B. Albrecht, E. Alguire, B. Austin, G. J. O. Beran, Y. A. Bernard, E. Berquist, K. Brandhorst, K. B. Bravaya, S. T. Brown, D. Casanova, C.-M. Chang, Y. Chen, S. H. Chien, K. D. Closser, D. L. Crittenden, M. Diedenhofen, R. A. DiStasio Jr., H. Dop, A. D. Dutoi, R. G. Edgar, S. Fatehi, L. Fusti-Molnar, A. Ghysels, A. Golubeva-Zadorozhnaya, J. Gomes, M. W. D. Hanson-Heine, P. H. P. Harbach, A. W. Hauser, E. G. Hohenstein, Z. C. Holden, T.-C. Jagau, H. Ji, B. Kaduk, K. Khistyaev, J. Kim, J. Kim, R. A. King, P. Klunzinger, D. Kosenkov, T. Kowalczyk, C. M. Krauter, K. U. Lao, A. Laurent, K. V. Lawler, S. V. Levchenko, C. Y. Lin, F. Liu, E. Livshits, R. C. Lochan, A. Luenser, P. Manohar, S. F. Manzer, S.-P. Mao, N. Mardirossian, A. V. Marenich, S. A. Maurer, N. J. Mayhall, C. M. Oana, R. Olivares-Amaya, D. P. O'Neill, J. A. Parkhill, T. M. Perrine, R. Peverati, P. A. Pieniazek, A. Prociuk, D. R. Rehn, E. Rosta, N. J. Russ, N. Sergueev, S. M. Sharada, S. Sharmaa, D. W. Small, A. Sodt, T. Stein, D. Stück, Y.-C. Su, A. J. W. Thom, T. Tsuchimochi, L. Vogt, O. Vydrov, T. Wang, M. A. Watson, J. Wenzel, A. White, C. F. Williams, V. Vanovschi, S. Yeganeh, S. R. Yost, Z.-Q. You, I. Y. Zhang, X. Zhang, Y. Zhou, B. R. Brooks, G. K. L. Chan, D. M. Chipman, C. J. Cramer, W. A. Goddard III, M. S. Gordon, W. J. Hehre, A. Klamt, H. F. Schaefer III, M. W. Schmidt, C. D. Sherrill, D. G. Truhlar, A. Warshel, X. Xua, A. Aspuru-Guzik, R. Baer, A. T. Bell, N. A. Besley, J.-D. Chai, A. Dreuw, B. D. Dunietz, T. R. Furlani, S. R. Gwaltney, C.-P. Hsu, Y. Jung, J. Kong, D. S. Lambrecht, W. Liang, C. Ochsenfeld, V. A. Rassolov, L. V. Slipchenko, J. E. Subotnik, T. Van Voorhis, J. M. Herbert, A. I. Krylov, P. M. W. Gill, and M. Head-Gordon. Advances in molecular quantum chemistry contained in the Q-Chem 4 program package.

[S9] A. Moliterni, D. Altamura, R. Lassandro, V. Olieric, G. Ferri, F. Cardarelli, A. Camposeo, D. Pisignano, J. E. Anthony, C. Giannini, *Acta Cryst.* **2020**, *B76,* 427–435.
